# Anaerobic corrosion of steel wire by *Geoalkalibacter ferrihydriticus* under alkaline autotrophic conditions

**DOI:** 10.1128/aem.01848-24

**Published:** 2025-03-10

**Authors:** Daria G. Zavarzina, Natalia I. Chistyakova, Jaroslav Kohout, Alexandr Yu Merkel, Anna A. Perevalova, Denisa Kubaniova, Michail S. Chernov, Evgeny N. Frolov, Alexey L. Klyuev, Sergey N. Gavrilov

**Affiliations:** 1 Winogradsky Institute of Microbiology, Research Center of Biotechnology, Russian Academy of Sciences 54744 , Moscow, Russian Federation; 2 Faculty of Physics, Lomonosov Moscow State University 133937 , Moscow, Russian Federation; 3 Faculty of Mathematics and Physics, Charles University 138735 , Prague, Czech Republic; 4 Faculty of Biology, Lomonosov Moscow State University 133839 , Moscow, Russian Federation; 5 Faculty of Geology, Lomonosov Moscow State University 243774 , Moscow, Russian Federation; 6 Frumkin Institute of Physical Chemistry and Electrochemistry, Russian Academy of Sciences 54744 , Moscow, Russian Federation; Washington University in St. Louis, St. Louis, Missouri, USA

**Keywords:** microbially induced corrosion, alkaline environments, iron-cycling anaerobes, *Geoalkalibacter ferrihydriticus*, steel, direct Fe^0^-oxidation, protons reduction

## Abstract

Microbially induced corrosion (MIC), caused by iron-cycling microorganisms that directly uptake electrons from metallic iron, is a serious economic and environmental problem. Iron corrosion is inhibited at pH above 9.0 in the presence of carbonate by the formation of a passivating film, but the possibility of direct oxidation of metallic iron by anaerobic alkaliphiles has not been thoroughly investigated. This bioinduced process may pose a serious environmental hazard under anaerobic alkaline conditions of underground radioactive waste disposal in metal containers with bentonite clays. We used *Geoalkalibacter ferrihydriticus*, an anaerobic iron-cycling bacterium capable of both dissimilatory iron reduction and anaerobic iron oxidation, as a model organism to investigate the microbial ability to utilize Fe^0^ from steel wire as an electron donor under anaerobic autotrophic conditions at pH 9.5. During bacterial growth, corrosion of the steel wire was induced and accompanied by intense H_2_ production and precipitation of a solid phase. Mössbauer spectroscopy revealed that green rust with siderite admixture was the major mineral formed during Fe oxidation. Protons appeared to be the only thermodynamically favorable electron acceptor for *G. ferrihydriticus*. Their reduction could lead to hydrogen production. Genomic analysis supported the proposal of such a metabolic mode for the organism. Thus, we have shown that MIC can be realized under anaerobic alkaline conditions by iron-cycling microorganisms in the absence of organic substrates. Microbial hydrogen production may facilitate the further development of authigenic microflora, which could further increase corrosion in radioactive waste repositories and reduce the barrier properties of bentonite clays.

**IMPORTANCE:**

Microbially induced corrosion (MIC) is a problem with significant economic damage. MIC processes occurring under anaerobic conditions at neutral pH have been actively studied over the last decades. Meanwhile, MIC processes under anaerobic alkaline conditions remain very poorly understood, although they represent a serious environmental problem, as such conditions are characteristic of the geological disposal of nuclear waste stored in metal containers isolated by clays. Our studies of the corrosion of steel by the anaerobic iron-cycling bacterium *Geoalkalibacter ferrihydriticus* at pH 9.5 in the absence of any organic matter have shown that this process is possible and can be accompanied by the active release of hydrogen. The formation of this gas can trigger the development of an authigenic anaerobic microflora that uses it as an electron donor and can negatively affect the insulating properties of the clay barrier through microbial metabolic activity.

## INTRODUCTION

Corrosion of iron presents a serious economic and environmental problem. Whereas aerobic corrosion is mainly a chemical process, corrosion of iron occurring under anoxic conditions is mostly caused by microbial metabolic activity (1). Microbially induced corrosion (MIC) or biocorrosion is the result of interactions between the metal surface, abiotic corrosion products, and microbial cells. It has several manifestations, the most important of which are local changes in the electrochemistry at the metal-solution interface (2, 3).

Today, different microbial groups, as well as their metabolites (4, 5) and enzymes (6, 7), are identified to be corrosion-active. Pioneering investigations of MIC at anaerobic conditions were focused on the activities of sulfate-reducing bacteria (SRB), producing hydrogen sulfide as a corrosive agent (8, 9). Later on, active cathodic depolarization of steel caused by microbial hydrogen consumption was demonstrated for SRB (9–11), methanogenic archaea (1, 12–15), acetogenic bacteria (16–18), and nitrate-reducing bacteria (19). However, recent studies revealed that only microorganisms capable of extracellular electron transfer (EET), which allows them to uptake electrons directly from metallic iron, can stimulate the cathodic reaction at the steel surface and thus enhance iron corrosion, while microbial H_2_ consumption alone is not sufficient to induce intense iron corrosion processes (1, 12, 18, 20, 21). EET has been extensively studied primarily in iron-cycling microorganisms that perform redox transformations of iron minerals during their use as energy sources or electron acceptors. It was shown that Fe(III)-reducing or Fe(II)-oxidizing organisms realize two major mechanisms of direct electron exchange with solid substances—via individual multiheme *c*-type cytochromes and their chains (22, 23) or via electrically conductive pili (24). Tang et al. (21) reported that the model Fe(III)-reducing bacterium *Geobacter sulfurreducens* can directly oxidize Fe^0^ as the sole electron donor using fumarate as the electron acceptor, and that outer surface *c*-type cytochromes OmcS and OmcZ are involved in this process.

It is well known that corrosion is pH-dependent. Iron corrosion at pH values over 9.0 is inhibited by the formation of ferrous passivating films. These films are usually composed of hydroxides, but in the presence of anions that yield less-soluble ferrous salts, such as carbonate, passivating films may also contain siderite (25, 26). These chemical conditions could be realized in natural alkaline anaerobic environments, such as soda lakes, characterized by highly diverse microbial communities (27). To date, alkaliphilic organisms have been identified among all corrosion-active metabolic groups of prokaryotes, such as sulfido-, methano-, and acetogens, as well as hydrogenotrophs and iron reducers (27–29).

Alkaliphilic corrosion-inducing microorganisms can destruct passivating films on the steel surface at high pH, which can be critical for steel containers used for underground disposal of nuclear wastes. The design of these facilities includes the use of cementitious construction and backfill materials, and the presence of metal in the construction (30). The cementitious materials employed in backfilling and construction generate alkaline environments up to pH 11 (31, 32). Such conditions favor the development of alkaliphiles, which can activate both negative (MIC, damage to the structural integrity of the host rock) and positive (nitrate reduction, cementation, metal reduction) processes in such geological disposals (33–35). In particular, nitrate and iron reduction decrease the redox potential of the environment due to the formation of reduced compounds that provokes reductive precipitation of radionuclides and their immobilization within the host rock of a disposal facility or on the surface of reduced iron minerals (36, 37).

It must be recognized that information on MIC under alkaline conditions is limited. There are only a few reports describing the ability of iron-reducing bacteria and SRB to promote the corrosion of carbon steel under alkaline conditions (38, 39).

The aim of current work is to demonstrate the ability of anaerobic alkaliphilic bacterium *Geoalkalibacter ferrihydriticus*, capable of Fe(III) reduction and Fe(II) oxidation via EET (40, 41), to induce corrosion of steel in carbonate-bicarbonate buffered medium at pH 9.5 during autotrophic growth.

Here, we report that the process of MIC could be enhanced by autotrophic microorganisms under alkaline anaerobic conditions directly by the EET mechanism with protons of water serving as the most probable inorganic electron acceptors. Genomic determinants of anaerobic iron corrosion coupled to hydrogen production were revealed in *G. ferrihydriticus* genome, and the main products of corrosion induced by *G. ferrihydriticus* were identified.

## MATERIALS AND METHODS

### Experimental methods

In this work, we used an active culture of the alkaliphilic iron-cycling bacterium *Geoalkalibacter ferrihydriticus* Z-0531^T^ (= VKM B-2349 = DSM 17461^T^) which we have previously isolated from the bottom deposits of the soda lake Khadyn (Tuva, Russia) with water pH 9.5 and total dissolved solids of 17.0 g/L (42).

Bundles of steel wire sponge (Emil Lux GmbH & Co. KG, Germany) were used as corrosion coupons. Prior to use, the initial state of steel wires was characterized by X-ray diffraction (XRD), X-ray fluorescence (XRF), and Mössbauer spectroscopic (MS) analyses. To remove possible organic impurities (traces of metalworking fluids), the wire was immersed in a solution of acetone and 96% ethanol (1:1) and subjected to ultrasonic cleaning for 30 min at 40°C before use. Then, the wire was washed three times with distilled water, laid on filter paper for drying, and finally dried in a thermostatically controlled oven at 80°C for 10 min.

### Cultivation conditions

The experiments were performed using anaerobic medium prepared as described previously (42) with the following composition (g/L): KH_2_PO_4_ –0.2; MgCl_2_×6H_2_O –0.1; NH_4_Cl – 0.5; KCl – 0.2; NaCl – 1.0; Na_2_CO_3_ – 3.0; NaHCO_3_ – 10.0; yeast extract – 0.02; trace element solution (43) – 1 ml/L. The pH value of the medium after sterilization was 9.5. For the first transfer, the medium was dispensed under N_2_ flux by 20 mL portions into 50 mL glass flasks containing 100 mg of steel wire. For all subsequent transfers, the medium was dispensed in 40 mL portions into 120 mL glass flasks containing 100 mg of steel wire. The medium was heat sterilized at 120°C for 30 min.

### Corrosion tests

For the inoculum in the corrosion tests, *G. ferrihydriticus* was grown on the medium, containing synthesized ferrihydrite [SF, 45 mM Fe(III)] as the electron acceptor and formate (10 mM) as the electron donor. As *G. ferrihydriticus* colonized the surface of the minerals, the cultures were strongly agitated before transfers, and after complete precipitation of the mineral phase, culture supernatants were used as inocula (5 vol %). Cell numbers of *G. ferrihydriticus* in the supernatants comprised ca. 2.5 × 10^6^ cell/mL. The inoculated flasks were incubated at 35°C for 120 days. After this first transfer, three subsequent transfers were performed on the same medium with steel sponge; each transfer was incubated for 180 days. The last transfer was used for the experiments with the addition of synthesized magnetite (0.1 g/L) or sodium sulfide (0.1 g/L).

Four variants of abiotic controls were used in the experiments: (1) a steel wire sponge in sterile anaerobic medium; (2) a steel wire sponge in sterile aerobic medium; (3) a single steel wire unraveled from the sponge and wrapped around a thin platinum bar in sterile anaerobic medium; (4) a steel wire sponge in sterile anaerobic medium containing sodium sulfide (0.1 g/L). Control flasks were incubated simultaneously with the cultures at the same temperature. The control (3) with the platinum bar was used to simulate electrochemical corrosion to compare its rate with MIC. All the experiments and controls were performed in triplicate.

### Analytical methods

Concentrations of H_2_ and acetate were monitored during all the incubation periods. After the end of the experiments, cell yields of *G. ferrihydriticus* were rated by routine q-PCR analysis; steel wire and solid precipitate formed during the experiments were analyzed by XRD and MS analyses and scanning electron microscopy (SEM).

Biofilms of *G. ferrihydriticus* on the surface of steel wires were visualized using a TCS SP5 scanning confocal laser microscope (Leica, Germany). Subsamples of the steel sponge were taken out from the grown cultures in the anaerobic glove box with sterile tweezers and scissors, put into 1.5 mL microcentrifuge tubes, and routinely stained with SYBR Green DNA stain (Invitrogen, USA) for further visualization under the confocal microscope at 561 nm laser wavelength.

The morphology of steel wires before and after incubation as well as the morphology of the precipitate formed during bacterial growth was studied by SEM on TESCAN VEGA 3 LMU microscope (OXFORD Instruments NanoAnalysis, UK). Samples were prepared by 2.5% glutaraldehyde fixation with ethanol dehydration and fixation on a conductive tape. To eliminate the charging effect, they were coated with gold. The samples were studied in the secondary electrons’ mode, with an accelerating voltage of 30 kV.

Samples of steel sponge and mineral precipitate for solid phase analysis were separated from the liquid phase, washed three times with acetone followed by centrifugation at 200 *g* for 5 min, dried under 100% N_2_, and then sealed into glass ampules under vacuum. Mineral composition was analyzed using an X-ray diffractometer EMPYREAN (PANalytical) with Cu Kα radiation, λ = 1.5405 Å, at room temperature in Bragg-Brentano geometry with a Pixel3D two-dimensional detector and a variable slit system. HighScore Plus (PANalytical) package with the ICSD PDF2 crystallographic database was used. Mössbauer spectroscopy was applied in accordance with the following protocol. The transmission MS experiments of samples were accomplished with a ^57^Co/Rh source using a conventional constant acceleration spectrometer by WissEL, Germany, and MS-1101Em spectrometer by Research Institute of Physics, Southern Federal University, Russia. Calibration of isomer shifts and the velocity scale was related to a standard α-Fe foil at 296 K. The SpectrRelax software (44) was used to analyze the Mössbauer spectra. The chemical composition of initial steel wire was determined by XRF analysis with an Eagle III µ-Probe spectrometer (Edax Inc., USA) equipped with Rh tube.

Subsamples of the gas phase for molecular hydrogen content monitoring were taken anaerobically with needles and syringes and analyzed using a gas chromatograph Crystal 5000.2 (Chromatech, Russia) equipped with a TCD detector and a Molecular Sieve 5A column (1 m), with extra pure argon as a carrier gas. Volatile fatty acids were determined on the same chromatograph equipped with a flame ionization detector (FID) and the following column: 0.3% Carbowax 20 M and 0.1% H_3_PO_4_ on Carbopack C 60–80 mesh (Supelco, USA). The cultures were sampled for volatile fatty acids using anaerobic technique with needles and syringes. Samples for chromatography were pre-treated by centrifugation at 12,600 *g* for 3 min followed by acidification of the clear supernatant with formic acid (extra pure grade) to pH 2.0.

### DNA extraction and q-PCR analysis

Primers for q-PCR-based quantification of 16S rRNA gene copies of *G. ferrihydriticus* Z-0531 were designed using Primer-BLAST software (45) with complete 16S rRNA gene sequences of *G. ferrihydriticus* as a target, and the rest of its genome as well as complete genome sequences of many other prokaryotes as an outgroup. The resulting primer system was GeoFer_532F (5′-GGACCGATACTGACGCTGAG-3′) and GeoFer_829R (5′-AGCCGAACTGACCCTCCTAT-3′). Amplicon size – 298 bp. Primers were used in 0.5 µM concentration. The PCR mixture was incubated for 5 min at 95°C, followed by 40 cycles of 20 s at 95°C, 20 s at 62°C, and 25 s at 72°C. DNA from cultures was isolated using FastDNA Spin Kit for Soil according to the manufacturer’s protocol (MP Biomedicals, Santa Ana, California, USA). Entire contents of the flask, i.e., liquid medium, steel wire, and solid precipitate formed during bacterial growth, were used for total DNA isolation. Genomic DNA of ferrihydrite-grown *G. ferrihydriticus* Z-0531 cultures was used for calibration curve. The cell content of the cultures used for calibration was determined by direct cell counting in preparations prestained with acridine orange dye using an Axio Lab.A1 fluorescence microscope (Zeiss, Germany). All reactions were carried out in triplicate. Correlation coefficient for the calibration curve (*R*^2^) was 0.997; PCR efficiency was 87%.

### Genome analysis

*G. ferrihydriticus* genome retrieved from DOE JGI IMG database (genome ID 2599185148) was screened for the genes of iron metabolism with a bioinformatics tool FeGenie (46) and manually against a set of ca. 70 previously described genes of multiheme cytochromes involved in dissimilatory Fe(III) reduction and energy-generating Fe(II) oxidation as well as the genes of related porin and pilin proteins (according to reference 47).

Hydrogenases were manually screened in the genome; query sequences were retrieved from public databases according to the data presented in reference 48.

### Thermodynamic calculations

The standard Gibbs free energy changes of the anodic and cathodic reactions were calculated under standard conditions (according to reference 49), then standard electrode potentials were calculated, and then the electrode potentials of the anodic and cathodic reactions under the experimental conditions (pH = 9.5, [HCO_3_^-^] = 0.119 M, and p(H_2_) = 1 atm) were determined by the Nernst equation. The equilibrium concentration of bicarbonate was calculated from the data on the content of the culture medium. The Gibbs free energies of formation used for the calculations are given in Table S1.

## RESULTS

### Steel wire composition

**Fig 1:**
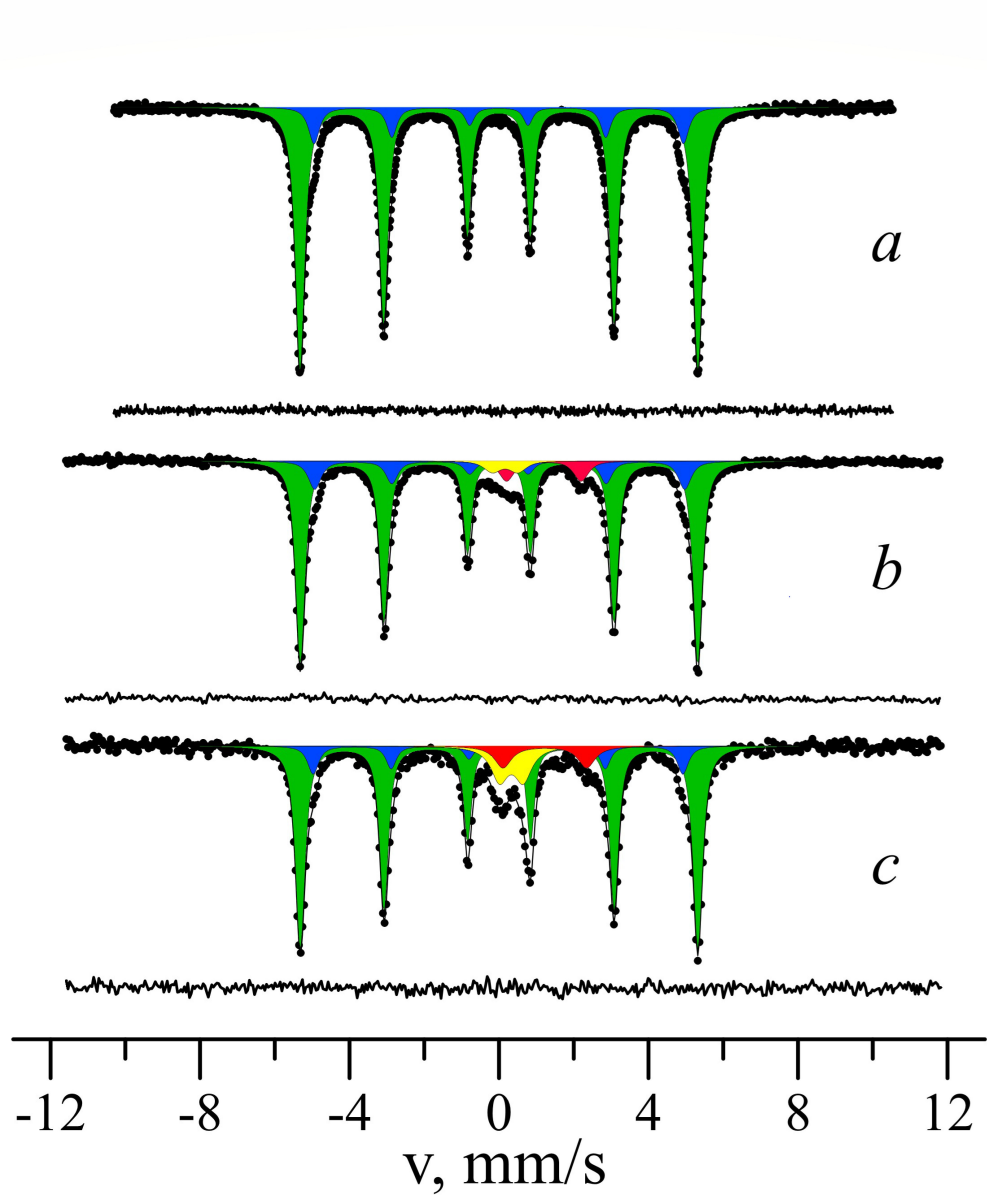
^57^Fe Mӧssbauer spectra of (**a**) initial “high strength alloy” steel wire sponge; (**b**) sterile anaerobic control after 180 days of incubation; (**c**) sterile anaerobic control with a platinum bar after 180 days of incubation. The subspectra corresponding to iron atoms in Fe_1-x_Mn_x_ structure without any Mn atoms in the nearest environment are marked green; the subspectra of Fe atoms with one Mn atom in the nearest environment are marked blue; and the subspectra corresponding to Fe^3+^ and Fe^2+^ ions in green rust structure are marked yellow and red, respectively.

XRF analysis (Fig. S1) revealed the wires to be the solid solutions of α-Fe and Mn with the calculated lattice parameter *a* = 2.881 ± 0.005 Å. XRD analysis supported the absence of iron mineral admixtures in the initial wire (Fig. S2a). Mössbauer spectrum of initial steel wires was collected at room temperature (Fig. 1). The fitting model consists of two Zeeman sextets with different relative intensities. The parameters of the subspectra are presented in Table S2. Since the sample is the solid solution of α-Fe and Mn, the presence of two sextets in the Mössbauer spectrum can be explained by the different nearest environments of the iron atoms in the structure. The parameters of the subspectrum S1 (*I*_1_ = 87.1 ± 0.8%) correspond to the parameters of the Mössbauer spectrum of ^57^Fe nuclei in the structure of α-Fe without impurity atoms in the nearest environment. The parameters of the subspectrum S2 (*I*_2_ = 12.9 ± 0.8%) correspond to the parameters of iron atoms with one manganese atom in the nearest environment. The probability of such a situation was determined using the binomial distribution. Using the value of the relative intensity *I*_2_ and the binomial distribution, the concentration of impurity atoms in the solution was estimated to be about 2.0 ± 0.1%. Therefore, the chemical formula of the solid solution in our case can be written as Fe_0.98_Mn_0.02_, which is close to a “high strength alloy“ steel content.

### Sterile controls

**Fig 2:**
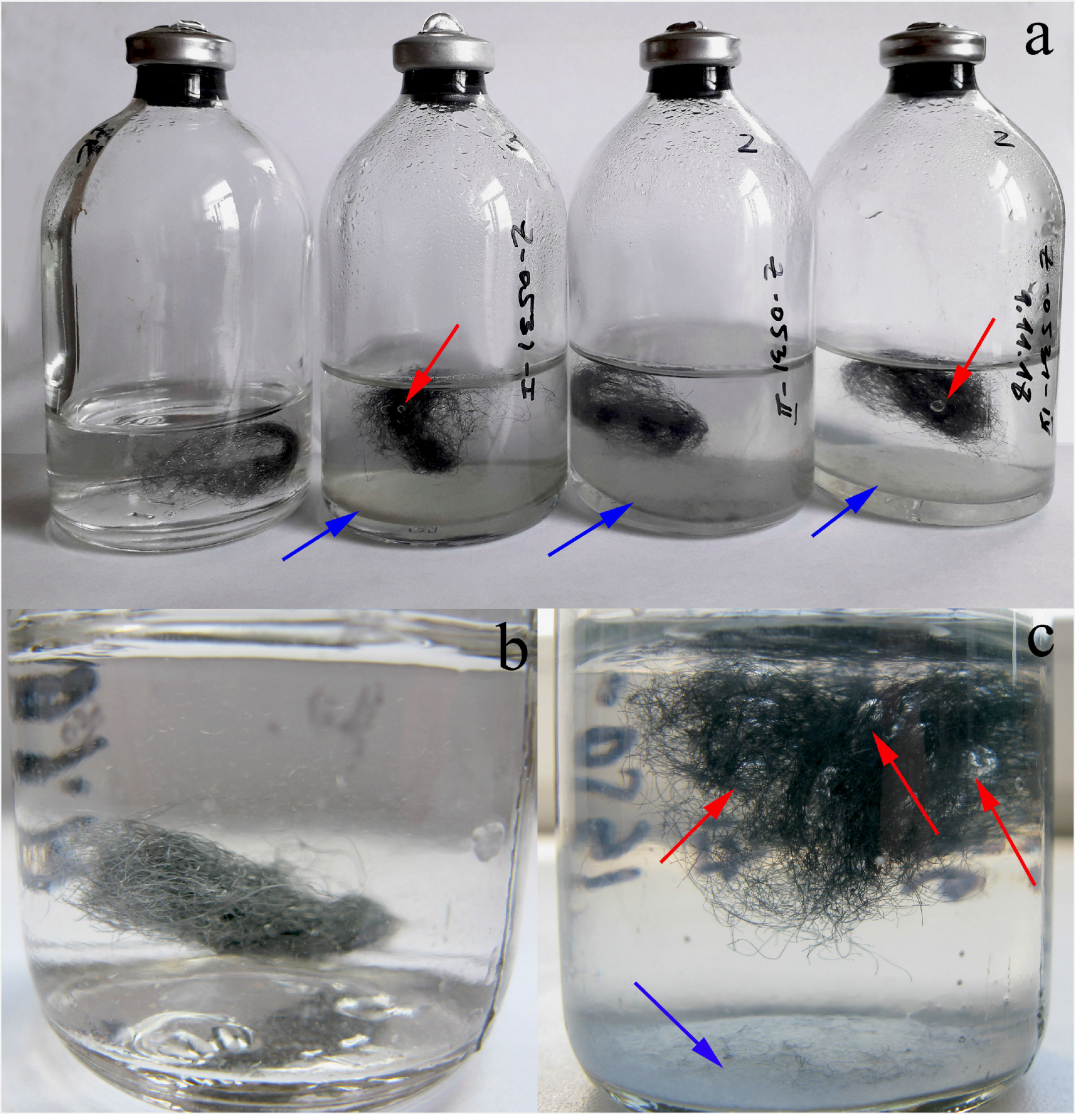
Change in the color of the steel wire accompanied by the formation of gas bubbles (red arrows [**a, c**]) and a precipitate (blue arrows [**a, c**]) after180 days of *G. ferrihydriticus* cultivation. Left flask at (**a**) magnified at (**b**)—sterile control; three right flasks at (**a**)—three subsequent transfers of *G. ferrihydriticus* culture.

No visual changes in the color and structure of the steel were observed in the anaerobic (Fig. 2) and aerobic sterile controls and in the control supplemented with synthesized magnetite (Fig. S3), while the control with a platinum bar showed an intense corrosion process accompanied by blackening of the steel wires and accumulation of rufous precipitate on the bottom of the flask (Fig. S3b). When sodium sulfide was added to the medium, the steel wire turned black immediately after sterilization and did not change throughout the incubation period (Fig. S3f). Gas phase analysis revealed that the H_2_ content fluctuated between 0.02 and 0.05 mM throughout the incubation period in the anaerobic and aerobic sterile controls and in the control supplemented with synthesized magnetite. In contrast, more than 3 H_2_ was formed by the end of the incubation in the sterile control with a platinum bar and more than 1.5 mM H_2_ was formed in the control with sodium sulfide (Table S3).

After 180 days of incubation of sterile controls, Mössbauer spectra were collected (Fig. 1b and c; Table S4). Two quadrupole doublets were added to the fitting model in comparison to the original steel wire spectrum. The parameters of the doublets D1 and D2 corresponded to ferrous and ferric atoms in newly formed phases, represented by green rust.

**Fig 3:**
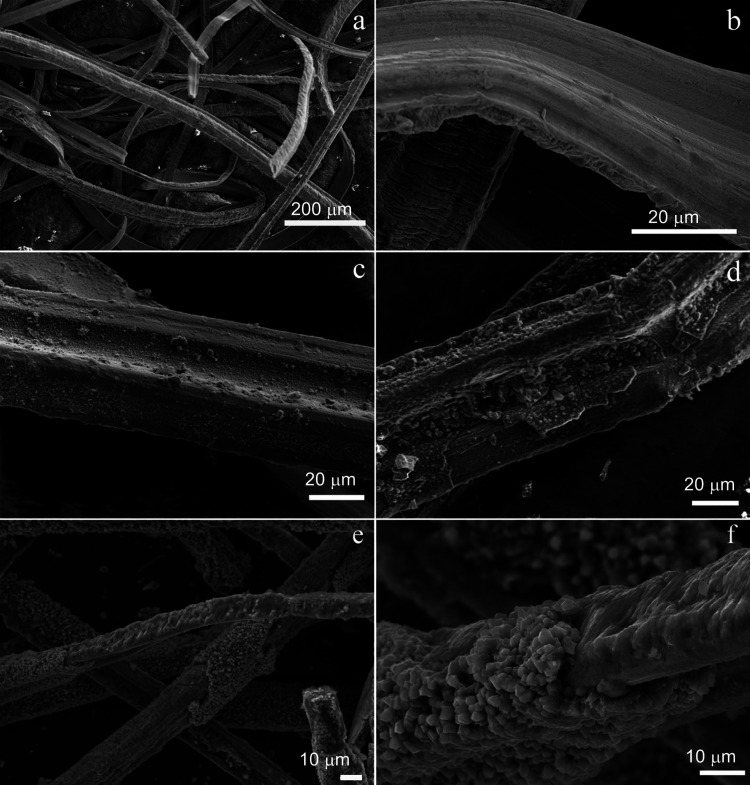
SEM micrographs of the steel wire: (**a, b**) different magnifications of the initial steel wire before the experiments; (**c**) steel wire in a sterile control after 180 days of incubation; (**d**) steel wire after 180 days of incubation in a *G. ferrihydriticus* culture; (**e, f**) different magnifications of the sterile control with platinum bar after 180 days of incubation.

SEM analysis of steel wire sponge in the sterile medium after incubation at aerobic and anaerobic conditions or with the addition of synthesized magnetite revealed minimal changes of steel surface morphology (Fig. 3c and d). The control with platinum was characterized by zonal corrosion of some wire parts (Fig. 3e and f).

### Growth of *Geoalkalibacter ferrihydriticus* with steel wire

#### First transfer culture

**Fig 4:**
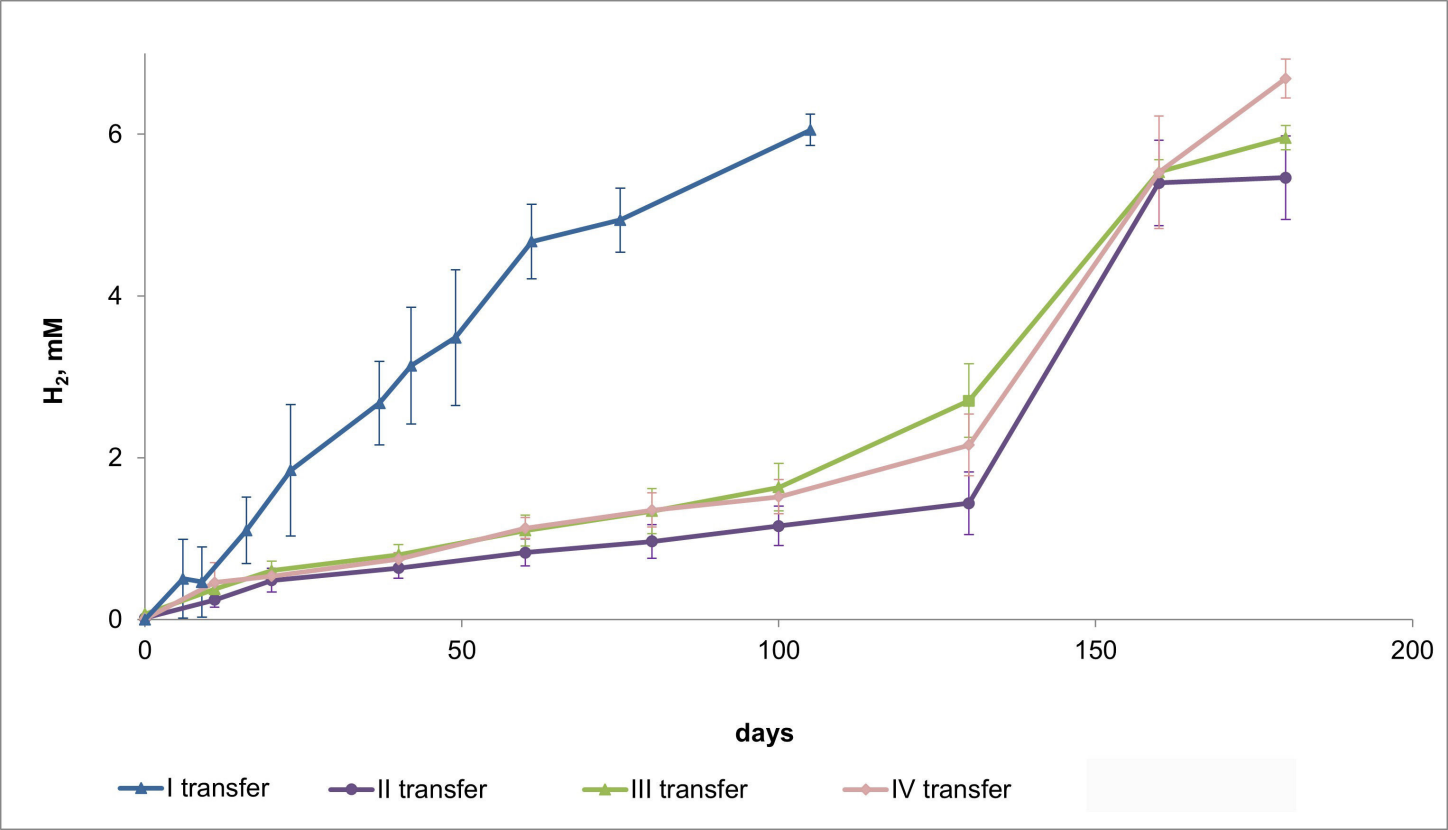
Production of H_2_ in *G. ferrihydriticus* cultures incubated with steel wire sponge in four subsequent transfers.

**Fig 5:**
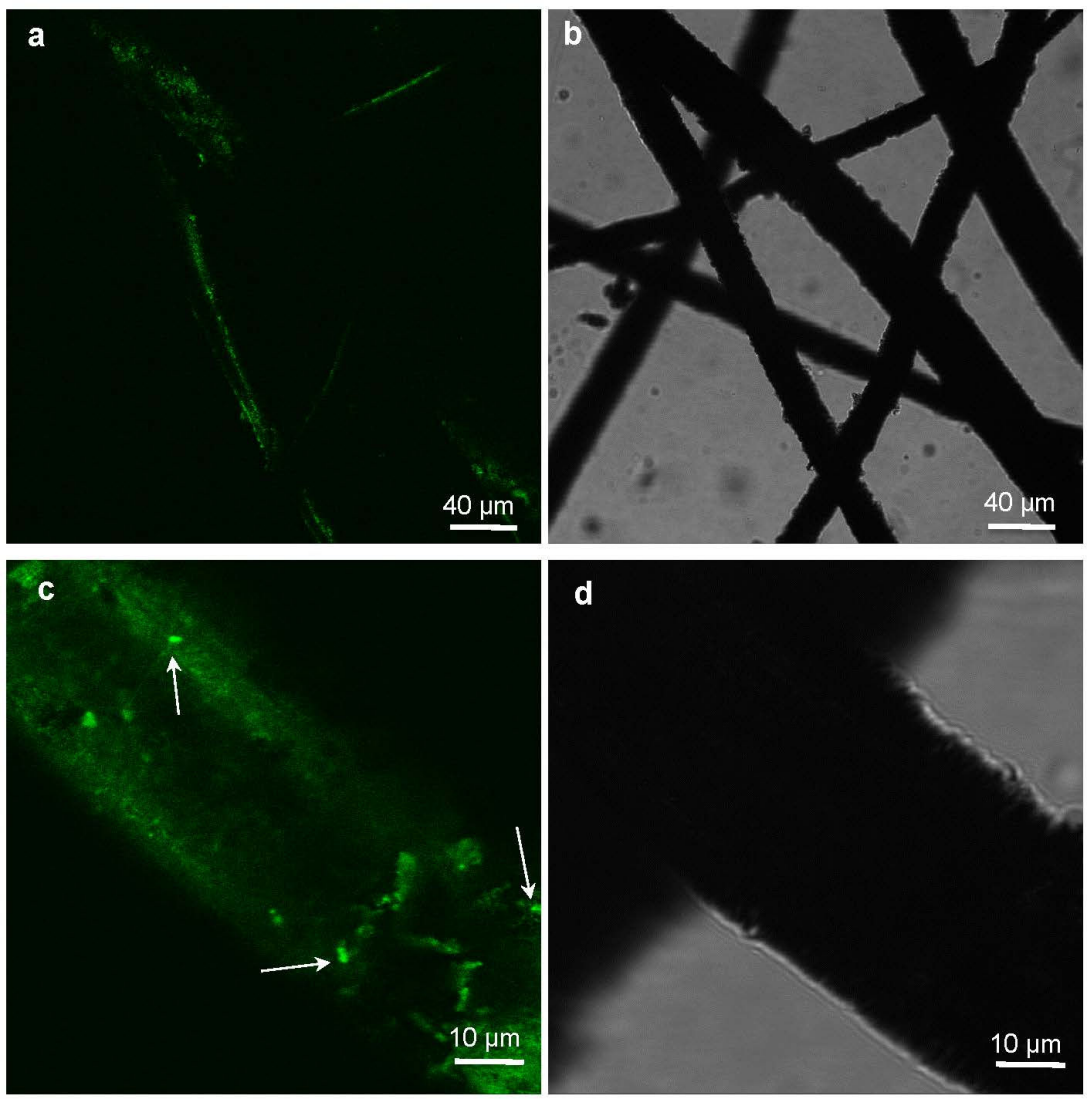
Confocal images of *G. sulfurreducens* biofilms on the surface of steel wires at two different spots taken at different magnification, spot 1 (**a and b**), spot 2 (**c and d**); (**a** and **c**)—fluorescent darkfield images; (**b** and **d**)—transmitted brightlight images. Arrows on (**c**) point clearly visible bacterial cells.

Anaerobic medium containing steel wire sponge was first inoculated with a *G. ferrihydriticus* culture grown with SF and formate (see Materials and Methods). Throughout the incubation period of this first transfer, the cell numbers of *Geoalkalibacter ferrihydriticus* in the medium did not exceed 5 × 10^5^ cells/mL according to q-PCR-based quantification, while the color of the steel wires gradually turned from metallic to black. After 50 days of incubation, the steel sponge has been covered with gas bubbles and surfaced to the top of the liquid medium. By this same time, thin bluish-green precipitate formed on the bottom of the flasks. The only detectable metabolic product formed during all the incubation period was H_2_, whose concentration reached 6.2 mM (millimole per liter of liquid culture) by the end of the experiment (Fig. 4). Laser confocal microscopy revealed almost complete fouling of the steel wire surface with *G. ferrihydriticus* cells. Uneven edges of microbial foulings and blurred profiles of individual cells on confocal micrographs indicate the formation of a multilayer biofilm with a polymeric matrix on the steel surface (Fig. 5).

**Fig 6:**
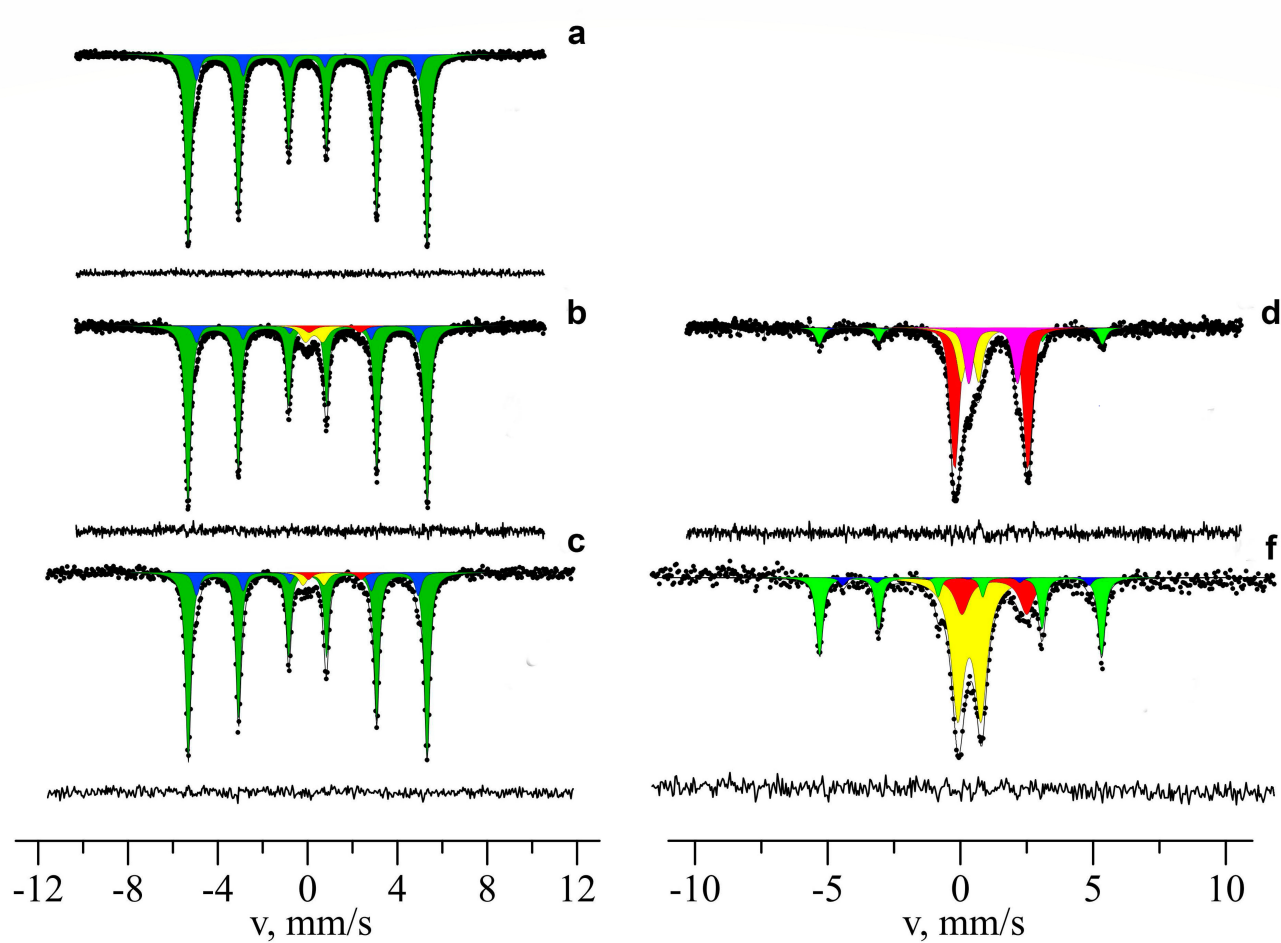
Mössbauer spectra of (**a**) the initial steel wire sponge; (**b**) steel wire after 120 days of incubation with *G. ferrihydriticus* (first transfer from a ferrihydrite-grown culture); (**c**) steel wire after 180 days of incubation with *G. ferrihydriticus* (fourth transfer from a ferrihydrite-grown culture); (**d**) precipitate formed after 120 days of incubation with *G. ferrihydriticus* (first transfer); (**f**) precipitate formed after 180 days of incubation with *G. ferrihydriticus* (fouth transfer). The subspectra corresponding to iron atoms in Fe_1-x_Mn_x_ structure without any Mn atoms in the nearest environment are marked green; the subspectra of Fe atoms with one Mn atom in the nearest environment are marked blue; the subspectra corresponding to Fe^3+^ and Fe^2+^ ions in green rust structure are marked yellow and red, respectively; and the subspectra corresponding to Fe^2+^ ions in siderite structure are marked pink.

The entire steel wire sponge and mineral precipitate were collected at the end of the incubation. Maximum sponge weight loss was 21% at the end of the first transfer culture incubation. Mössbauer studies of the steel wire and the precipitate after the end of incubation were carried out at room temperature (Fig. 6). The obtained spectra were fitted using a model consisting of two sextets (S1 and S2) and two quadrupole doublets (D1 and D2) (Fig. 6b). Parameters S1 and S2, as in the case of the spectrum of the initial wire, corresponded to the parameters of iron atoms in the structure of the solid solution. Parameters D1 and D2 corresponded to the parameters of ferrous and ferric atoms in the green rust structure (Table S5). In the case of the precipitate, an additional quadrupole doublet (D3) spectrum was added to the model, which refers to ferrous atoms in the structure of a newly formed phase other than green rust (Fig. 6d). The parameters of this subspectrum were similar to the parameters of ferrous atoms in the siderite structure (Table S5). XRD analysis supported the formation of (oxy)hydroxycarbonate iron minerals within the precipitate formed by the first transfer culture (Fig. S2b and c).

#### Stable iron-corroding cultures

Within three subsequent transfers of the “first transfer” culture on the same medium, each time supplied with fresh steel wire sponges, the corrosion rate slowed down but the cell yield and final production of molecular hydrogen remained stable (Tables S3 and S6). By the end of the experiments, in the fourth transfer with the sponge, 5.5–6.7 mM H_2_ formed in the flasks (Fig. 4). In all the cases, the steel sponges surfaced to the top of the medium and changed their color from metallic to black. Simultaneously, the formation of a bluish-green mineral precipitate on the bottom of the culture flasks was observed (Fig. 2a and c).

Quantitave PCR analysis demonstrated that cell numbers of *G. ferrihydriticus* in the cultures did not exceed 4 × 10^6^ cell/ml (Table S6).

**Fig 7:**
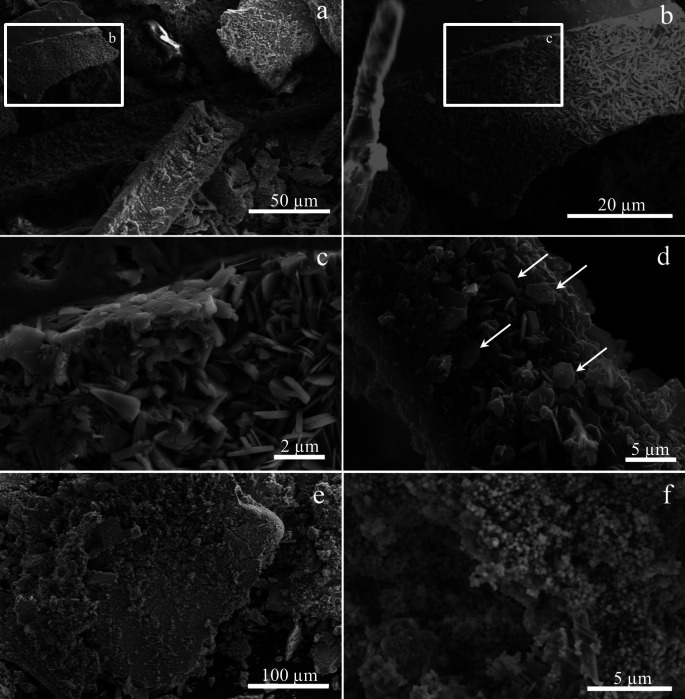
SEM micrographs of the steel wire and the precipitate after 180 days of incubation in *G. ferrihydriticus* culture: (**a–c**) different magnifications of the steel wire (**a, b**) and a precipitate (**c**) containing tabular crystals of green rust; (**d**) tabular crystals of green rust (marked by arrows) formed on the surface of the steel wire; (**e, f**) different magnifications of the precipitate containing spherical crystals (probably, siderite).

SEM analysis revealed identical changes in the structure of the steel surface and identical characteristics of the mineral precipitate in all the four transfers of the culture with the steel sponge (Fig. 7). The surface of the wires was intensely corroded and covered with hexagonal tabular crystals (Fig. 7c and d) typical of green rust (50). The mineral phase, precipitated during bacterial growth on the bottom of the flasks, was represented by a mixture of nano-sized globules (Fig. 7e and f) and archetypal green rust crystals. The weight loss of the steel wire after incubation in the second to fourth transfer cultures was twice as low as in the first transfer culture.

Confocal fluorescent microscopy revealed dense foulings of steel wires with microbial cells, whose blurred contours indicated the formation of thick biofilms with a high content of extracellular polymeric substances (EPS). Active production of EPS and the formation of dense biofilms on mineral surfaces have been previously reported for *G. ferrihydriticus* (41).

All Mössbauer spectra of biotransformed steel wire samples and the newly formed precipitate were processed using a single model. The fitting model consisted of two Zeeman sextets related to α-Fe and Mn solid solution, and two doublets related to ferrous and ferric atoms in the structure of a newly formed solid phase. The obtained hyperfine parameters of these subspectra are similar to the hyperfine parameters of iron atoms in the structure of green rust (Fig. 6c and f; Table S5).

#### Control experiments with the addition of synthesized magnetite or sodium sulfide

The addition of synthetic magnetite slightly increased the rate of hydrogen production at the beginning of the bacterial growth (Table S3). The rate increased up to the values observed during the first transfer of *G. ferrihydriticus* to the medium with steel wire sponge (Fig. 4). However, the final hydrogen concentration did not exceed that observed in the second to fourth transfers of the cultures lacking magnetite (Table S3). Mössbauer and SEM analysis revealed identical changes in the structure of the steel surface and identical characteristics of the mineral precipitate in both magnetite-supplemented and regular cultures with the steel sponge (Tables S4 and S5; Fig. S4a and b). The addition of sulfide to the culture medium resulted in a complete inhibition of *G. ferrihydriticus* growth. Throughout the control experiment with sulfide, the hydrogen concentration as well as the changes in the structure of the steel surface and characteristics of the mineral precipitate were almost identical to those of the sterile control without sulfide, indicating that microbial cells were not involved in the process of interaction between sulfide and metallic iron (Tables S3 to S5; Fig. S3e, f, S4c and d).

#### Genome analysis of *G. ferrihydriticus*

After manual curation of the results retrieved by the FeGenie bioinformatic tool (46), the analysis revealed a total of 42 genes which could determine EET processes in *G. ferrihydriticus*. In addition to previously described genes of key components of porin-cytochrome Fe(II)-oxidizing complexes (MtoA and MtoC) and an e-pilin (40), the genes of other 32 multiheme cytochromes with predicted 2 to 38 heme-binding sites were identified, as well as the genes of “long” type-IV pilins and cytochrome *b*-containing quinol-oxidizing complexes.

The majority of identified multiheme cytochromes were predicted to be secreted proteins, and several of them shared homology with the components of porin-cytochrome complexes determining the reduction of Fe(III) oxides in *Geobacter sulfurreducens* and *Shewanella oneidensis* (22, 51).

Most of multiheme-encoding genes are grouped in loci. The biggest locus (Ga0056053_02426–02431) encodes homologs of MtoA (Ga0056053_02428, previously described as Gfer_00954 [40]), a cytochrome sharing equally low homology with MtoA and MtoC (Ga0056053_02430), a putative quinol-oxidizing/reducing cytochrome Ga0056053_02431, a cytochrome homologous to OmcE cell surface protein of *Gbr. sulfurreducens*, and two proteins with NHL repeat domains. The latter proteins are functionally diverse and, in particular, could serve as the basis for mineral nanocrystals binding (52). Additionally, the closest homolog of MtoC protein, proposed to mediate electron transfer between the quinone/quinol pool and redox proteins in the periplasm (53), is encoded separately in *G. ferrihydriticus* genome by Ga0056053_01081 (Gfer_00569 as by [40]), downstream of several ATP synthase subunits and upstream of two predicted transmembrane cytochromes Ga0056053_01083 and Ga0056053_01086. This genomic neighborhood correlates with presumed involvement of Ga0056053_01081 in energy generation. Also, four genes of the proteins poorly homologous to MtoA are scattered along *G. ferrihydriticus* genome (Ga0056053_00187, Ga0056053_00321, Ga0056053_02460, and Ga0056053_03439).

Fe(III) reduction in *G. ferrihydriticus* is determined by several genomic loci. The most probable determinant encodes a protein Ga0056053_02659, equally homologous to MtrA, OmhA, and OcwA putative terminal Fe(III) reductases of *S. oneidensis*, *Carboxydothermus ferrireducens*, and *‘Thermincola potens’*, respectively. Another protein, encoded in this locus (Ga0056053_02660), shares homology with OmcB cytochrome of *Gbr. sulfurreducens*, regarded to stabilize its terminal Fe(III) reductase on the cell surface (54). Further candidate determinant of Fe(III) reduction in *G. ferrihydriticus* could be the locus Ga0056053_00179–Ga0056053_00187 encoding putative quinol oxidase and two dodecaheme secreted cytochromes, or the locus Ga0056053_01388–Ga0056053_01389 encoding a membrane bound and a secreted cytochrome with 14 and 13 heme-binding sites, respectively. Other multiheme cytochrome genes scattered along *G. ferrihydriticus* genome could also be involved in EET to insoluble Fe(III) oxides, and their roles in this process are to be evaluated. Among such cytochromes, the protein Ga0056053_03448 deserves special attention. This large (1,208 aa) protein contains 10 different conserved multiheme Pfam domains totally harboring 24 heme-binding sites, and two N-terminal fibronectin type III domains related to cell adhesion. Interestingly, this protein is encoded downstream of a larger, 38-heme cytochrome homologous to OmcV outer membrane multiheme of *Gbr. sulfurreducens*. Additionally, *G. ferrihydriticus* possesses a homolog of OmcZ octaheme (Ga0056053_00060), which has been reported to be dispersed throughout the extracellular matrix of *Gbr. sulfurreducens* and proposed to enhance EET (55, 56).

Besides a rich repertoire of multiheme cytochromes, genome of *G. ferrihydriticus* encodes three different type IV pili assemblies. One of the three loci encoding these extracellular structures contains the previously described putative conductive e-pilin Ga0056053_00657 (41). Our current analysis revealed another closely located genomic region Ga0056053_00633–645 encoding a putative pili assembly with a “long-type” IVa poorly conductive pilin Ga0056053_00641. Additionally, a locus Ga0056053_01527–1535 encoding a “PilA–C” (GspG) protein Ga0056053_01535 was identified in *G. ferrihydriticus*. Such proteins contain a C-terminus of similar topology to type IVa pilins and an N-terminus similar to porin-like proteins, and their function is yet unclear (57); however, a “PilA-C” homolog from *C. ferrireducens* was recently shown to be overexpressed by piliated Fe(III)-reducing cells (58). Interestingly, the “PilA-C” locus of *G. ferrihydriticus* also encodes two secreted multiheme cytochromes Ga0056053_01525 and Ga0056053_01526, which could be involved in EET along the pilin filaments in a way similar to OmcS cytochrome of *Gbr. sulfurreducens*, although this proposal needs further experimental evaluation.

Among other determinants of EET processes, a succinate dehydrogenase-encoding locus Ga0056053_01564–01566, a cytochrome *b*/*c* quinol oxidase-encoding locus Ga0056053_02118–2120, and the genes of Ndh2, FmnAB, EetAB, and DmkB complexes of a flavin-based EET mechanism (59) were identified in *G. ferrihydriticus*.

One of the most probable reactions for electron acceptance during the autotrophic growth of *G. ferrihydriticus* by Fe(0) oxidation could be the reduction of protons to molecular hydrogen. The organism possesses four gene clusters encoding putative hydrogen-evolving [NiFe] hydrogenases. Of them, one cluster (Ga0056053_02787–88) encodes a two-subunit cytoplasmic hydrogenase and three other clusters encode the subunits interacting with NAD(P)H as the cofactor, Fe-S subunits, NiFe catalytic subunits, and hydrogenase maturation factors. The cluster Ga0056053_00724–29 encodes six proteins HoxEFUYHM related to a cytoplasmic [NiFe] hydrogenase complex. Its most closely related biochemically characterized homolog (42.9% identity, Ev 3.9·10^−127^ for HoxH) is the NAD-reducing hydrogenase of the strain *Cupriavidus necator* H16 (formerly *Ralstonia eutropha*) which is proposed to have proton-reducing activity (60). Notably, putative HoxEFU subunits of this complex in *G. ferrihydriticus* share similarity with HydABC subunits of the electron-bifurcating hydrogenase of *Thermoanaerobacter kivui* (max. 48% identity, Ev 3.9·10^−177^ at query coverage 98% between HydB and Ga0056053_00725 proteins). Another gene cluster of *G. ferrihydriticus*, Ga0056053_02666–69, encodes a similar enzyme complex with a putative membrane-anchoring subunit. Its large subunit with the catalytic NiFe site (Ga0056053_02666–68) also possesses a membrane-anchoring domain. The most closely related biochemically characterized homolog of this protein is the catalytic subunit of an archaeal bifunctional enzyme complex from *Pyrococcus furiosus* (33.9% identity, Ev 10^−58^) that functions as an NADPH-dependent H_2_-evolving hydrogenase with sulfur-reducing activity (61). In addition, a large catalytic subunit of one else [NiFe] hydrogenase, encoded in *G. ferrihydriticus* by Ga0056053_02677, is homologous (42.9% identity, Ev 9.4·10^−138^) to a group 1 hydrogenase of *Nitratidesulfovibrio vulgaris* strain Miyazaki F, which could use a cytochrome *c_3_* as the electron-transferring partner for proton reduction or H_2_ oxidation (62). This same enzyme was shown to be a promising electrocatalyst uptaking electrons from a carbon felt electrode for proton reduction via an electron shuttle (63).

#### Thermodynamic calculations of Gibbs free energy changes for the reactions of steel wire oxidation under experimental conditions with protons of water as the electron acceptors

The anodic reaction of substrate oxidation, i.e., the electron source, with green rust as the product, written in a forward direction:


(1) $$6{Fe}^{\left(0\right)}+{HCO}_{3}^{-}+13{OH}^{-}+H_{2}O\to GR{+14e}^{-}$$

The anodic reaction written in the opposite direction according to the IUPAC Compendium of Chemical Terminology (49) to calculate ∆G and E correctly:


(2) $$GR{+14e}^{-}\to 6{Fe}^{\left(0\right)}+{HCO}_{3}^{-}+13{OH}^{-}+H_{2}O$$

(3) $$GR=\left[{Fe}_{4}^{\left(II\right)}{Fe}_{2}^{\left(III\right)}{\left(OH\right)}_{12}\right]\left[{CO}_{3}∙2H_{2}O\right]$$

The standard Gibbs free energy change of the anodic reaction with GR as the product:


(4) $$\begin{array}{c} \Delta G_{298,a}^{0}=\left(\Delta G_{f,298}^{0}\left(HCO_{3}^{-}\right)+13\Delta G_{f,298}^{0}\left(HCO_{3}^{-}\right)+\Delta G_{298}^{0}\left(H_{2}O\right)\right)-\Delta G_{f,298}^{0}(GR)=\\ =1195\;kJ\cdot {\;mole}^{-1} \end{array}$$

The standard electrode potential of the anodic reaction with GR as the product:


(5) $$E_{a}^{0}=-\frac{{∆G}_{298,a}^{0}}{n\times F}=-\frac{1195\times 10^{3}}{14\times 96480}=-0.885V$$

The electrode potential of the anodic reaction with GR formed under the experimental conditions:


(6) $$\begin{array}{rl} & E_{a}=E_{a}^{0}+\frac{0.059}{14}lg\frac{1}{\left[{HCO}_{3}^{-}\right]\cdot {\left[{OH}^{-}\right]}^{13}}=\\ & \;=-0.885-\frac{0.059}{14}lg\left[{HCO}_{3}^{-}\right]+\frac{13\times 0.059}{14}(14-pH)=-0.637\;V \end{array}$$

The cathodic reaction of water reduction, i.e., the electron sink:


(7) $$2H_{2}O{+2e}^{-}\to 2{OH}^{-}+H_{2}$$

The standard Gibbs free energy change of the cathodic reaction:


(8) $${∆G}_{298,c}^{0}=2\times {∆G}_{298}^{0}\left({OH}^{-}\right)-2\times {∆G}_{298}^{0}\left(H_{2}O\right)=159.6kJ\cdot {mole}^{-1}$$

The standard electrode potential of the cathodic reaction:


(9) $$E_{c}^{0}=-\frac{{∆G}_{298,c}^{0}}{n\times F}=-\frac{159.6\times 10^{3}}{2\times 96480}=-0.827V$$

The electrode potential of the cathodic reaction under the experimental conditions:


(10) $$E_{с}=E_{с}^{0}+\frac{0.059}{2}lg\frac{1}{{\left[OH^{-}\right]}^{2}}=-0.827+0.059\left(14-pH\right)=-0.059pH=-0.561\;V$$

The overall reaction with green rust as the product:


(11) $$6Fe^{\left(0\right)}+HCO_{3}^{-}+15H_{2}O\to \left[Fe_{4}^{\left(II\right)}Fe_{2}^{\left(III\right)}{\left(OH\right)}_{12}\right]\left[CO_{3}\cdot 2H_{2}O\right]+OH^{-}+7H_{2}$$

The total potential change under standard conditions:


(12) $$E^{0}=E_{c}^{0}-E_{a}^{0}=-0.827-\left(-0.885\right)=0.058V$$

The standard Gibbs free energy change of the overall reaction under standard conditions:


(13) $$\Delta G^{0}=-nFE^{0}=-14\times 96480\times 0.058=-78341\;J\cdot mole^{-1}=-78.3kJ\cdot mole^{-1}$$

The total potential difference in the reaction of green rust formation under experimental conditions:


(14) $$E=E_{c}-E_{a}=-0.561-\left(-0.637\right)=0.076\;V$$

The Gibbs free energy change of the overall reaction of green rust formation under experimental conditions:


(15) $$\Delta G=-nFE=-14\times 96480\times 0.076=-102654\;J\cdot mole^{-1}=-102.6\;kJ\cdot mole^{-1}$$

The anodic reaction of substrate oxidation with siderite as the product, written in opposite direction:


(16) $$Fe{CO}_{3}+H^{+}+2e^{-}\to Fe+{HCO}_{3}^{-}$$

The standard Gibbs free energy change of the anodic reaction with siderite as the product:


(17) $${∆G}_{298,a}^{0}={∆G}_{f,298}^{0}\left({HCO}_{3}^{-}\right)-{∆G}_{f,298}^{0}\left(Fe{CO}_{3}\right)=79.8kJ\cdot {mole}^{-1}$$

The standard electrode potential of the anodic reaction with siderite:


(18) $$E_{a}^{0}=-\frac{{∆G}_{298,a}^{0}}{n\times F}=-\frac{79.8\times 10^{3}}{2\times 96480}=-0.414V$$

The electrode potential of the anodic reaction with siderite under the experimental conditions:


(19) $$E_{a}=E_{a}^{0}+\frac{0.059}{2}lg\frac{\left[H^{+}\right]}{\left[HCO_{3}^{-}\right]}=-0.414-\frac{0.059}{2}lg\left[HCO_{3}^{-}\right]-\frac{0.059}{2}pH=-0.667\;V$$

The overall reaction with siderite as the product:


(20) $$Fe+{HCO}_{3}^{-}+H_{2}O\to Fe{CO}_{3}+{OH}^{-}+H_{2}$$

The total potential difference in the reaction of siderite formation under experimental conditions:


(21) $$E=E_{с}-E_{a}=-0.561-\left(-0.667\right)=0.106V$$

The Gibbs free energy change of the overall reaction of siderite formation under experimental conditions:


(22) $$\Delta G=-nFE=-2*96480*0.106=-20453\;J\cdot mole^{-1}=-20.5\;kJ\cdot mole^{-1}$$

## DISCUSSION

Biocorrosion in anoxic environments could be mediated by different groups of microorganisms in diverse circum-neutral environments. In addition to previously known pathways, microbial electron uptake directly from Fe^0^ has recently been recognized as one of the major causative factors of MIC. This process, specially termed electrical MIC (EMIC), was reported for several physiological groups of microorganisms, such as SRB, methanogens, acetogens, and nitrate- and iron-reducing bacteria (3, 20, 21). The presence of microorganisms inducing EMIC in alkaline environments can impact some crucial economic activities. For instance, nuclear waste duplex stainless steel containers in concrete bunkers can be subjected to MIC from high pH water percolating through the concrete (39, 64). Besides, microbial metabolic activity could impact bicarbonate and carbonate concentrations, which are considered to be among the most critical and widespread electroactive species inducing corrosion in oil and gas pipelines (26) and the geological disposal of high- and intermediate-level nuclear waste storage (30). However, no particular organisms have been reported so far to perform EMIC at high pH.

The choice of *G. ferrihydriticus* as the microbial agent for the study of MIC in an alkaline environment was determined by the ability of the representatives of this genus to realize both the reduction and oxidation of insoluble iron minerals or electrodes during their anaerobic growth (40–42, 65–69). That means *G. ferrihydriticus* can realize either outward or inward extracellular electron transfer, depending on the cultivation conditions, and thus can promote cathodic depolarization or take electrons directly from Fe^0^.

The results of our experiments clearly show the ability of *G. ferrihydriticus* to promote corrosion of steel wire. The transformation of the steel, accompanied by active H_2_ gas formation, resulted in the accumulation of oxidation products of Fe^0^, which were deposited on the steel surface as well as separately of it, on the bottom of the cultivation flasks (Fig. 2, 3, and 7). To identify the green rust varieties (GR) formed as the main corrosion products, we calculated the total stoichiometry parameter (*R*_summ_) from Mössbauer data considering both the GR formed on the steel wire (Fig. 6b and c) and the GR precipitated separately from the wire (Fig. 6d and f) according to the following equation: *R*_summ_ = (*I*_w3+_ + *I*_s3+_)/(*I*_w3+_ + *I*_s3+_+ *I*_w2+_+ *I*_s2+_), where *I*_w2+_ and *I*_w3+_ are the relative intensities of the quadrupole doublet corresponding to Fe^2+^/Fe^3+^ ions in the Mössbauer spectrum of the GR in the wire, and *I*_s2+_ and *I*_s3+_ are the intensities from the spectrum of GR in the precipitate. The *R*_summ_ value in the first transfer culture (Fig. 6b; Table S5) was the smallest, equal to 0.34 ± 0.01. The obtained value of the stoichiometry parameter *R*_summ_ allows us to conclude that, in this case, fougèrite [Fe^2+^_4_Fe^3+^_2_(OH)_12_][CO_3_]·3H_2_O was the main mineral of GR. The precipitate obtained in the first transfer culture was significantly different from subsequent ones. In addition to GR, a large amount of siderite (about 20%) was detected in this precipitate (Fig. 6d; Table S5). In the cultures obtained after four successive transfers with the steel wire, no siderite was formed and the stoichiometry parameter for GR was of higher value, being practically similar for the second, third, and fourth transfers (0.73 ÷ 0.77 ± 0.03). It can be assumed that subsequent transfers of the culture led to the production of a mixture of trébeurdenite [Fe^2+^_2_Fe^3+^_4_O_2_(OH)_10_][CO_3_]·3H_2_O and mössbauerite Fe^3+^_6_O_4_(OH)_8_[CO_3_]·3H_2_O (70). Formation of carbonaceous minerals rather than oxides is the corollary of the presence of carbonate and bicarbonate anions in a high pH medium, where they form less-soluble Fe salts than hydroxyl anions (25, 26, 38, 71). Thus, the formation of Fe(III)-containing GR as the main product of steel wire corrosion in anaerobic medium in our experiments is not surprising. For example, Lee et al. (38) reported the importance of green rust in stabilizing the corrosion rates in de-oxygenated 0.2 M NaHCO_3_ + Na_2_CO_3_ solutions. In more concentrated solutions, they reported that carbonate-complexed Fe^2+^ ions make the FeCO_3_·H_2_O films increasingly more porous, thereby increasing corrosion rates (38).

**Fig 8:**
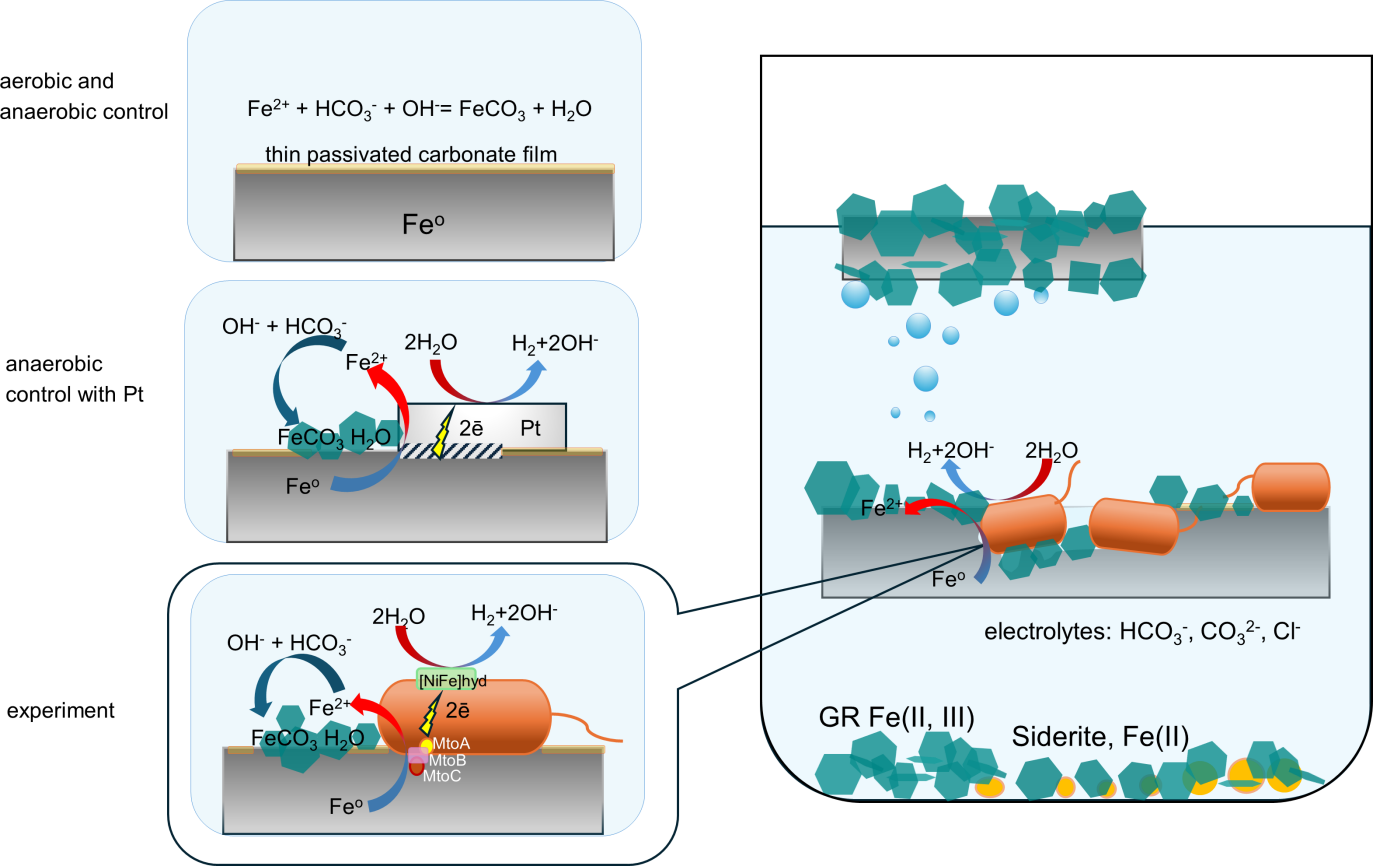
Schematic representation of the proposed corrosion processes induced by *G. ferrihydriticus*.

In our experiments, the rate and intensity of the corrosion process and the production of hydrogen in the growing cultures were much higher than in the sterile controls, with the exception of the control with a platinum bar (Table S3). In the latter case, the heterogeneity in the composition of the steel wire due to the presence of manganese admixture caused the galvanic couple to appear at the contact of platinum with the steel, resulting in electrochemical corrosion accompanied by the formation of green rust and the production of hydrogen (Fig. 8; Fig. S3b; Table S4). The addition of sodium sulfide to the medium with the steel wire had a dramatic effect on the growth of *G. ferrihydriticus*, completely inhibiting it. It is well known that sulfide reacts with metallic iron to form a passivating FeS film (3, 9), which is more stable under alkaline than under neutral conditions. This film could act as an insulator for electrochemical interactions of *G. ferrihydriticus* cells with steel. Accordingly, the inhibitory effect of sulfide and the opposite stimulating effect of synthesized magnetite on *G. ferrihydriticus* growth and hydrogen production indicate that the organism uses metallic iron as an electron donor by the EET mechanism, either directly or through a conductive solid mediator such as magnetite.

SEM studies did not visualize any clear cell-like structures on steel wire surfaces. However, fluorescent confocal microscopy revealed biofilm-like colonization of sponge wires by *G. ferrihydriticus*, which hampered the growth monitoring by direct cell counting (Fig. 5). The results of q-PCR analysis indicated that cell numbers of *G. ferrihydriticus* increased, at least, an order of magnitude during the incubation period (Table S6). The most intriguing question about the observed growth is: What chemical reaction supported it? Steel wires could be affected by microbial growth and metabolic activity in two scenarios, i.e., hydrogenotrophic reduction of electrochemically produced Fe(III) or direct metabolic oxidation of Fe^0^ with protons as electron acceptors.

If the first scenario were realized in our experiments, *G. ferrihydriticus* would use ferric iron from green rust as the electron acceptor. In such a case, cathodic reaction leading to H_2_ formation would provide the bacterium with an electron donor, and siderite would be the product of green rust reduction by *G. ferrihydriticus*. We observed the formation of siderite only in the first transfer culture, where a small amount of magnetite or synthesized ferrihydrite may have appeared as an admixture of inoculum (Fig. 6b and d; Table S5). However, a steady increase of H_2_ concentration throughout the cultivation period in our experiments doubts the realization of hydrogenotrophic Fe(III) reduction, despite the identification of a rich repertoire of genes determining the outward EET in *G. ferrihydriticus*. We suggest that *G. ferrihydriticus* more likely utilizes Fe^0^ as the electron donor via direct inward EET mechanism, thus producing oxidized iron in the form of green rust with siderite admixture. The ability of this bacterium to oxidize iron at anaerobic conditions has been previously reported in the experiments with siderite (40). Genome analysis of *G. ferrihydriticus* within the current study revealed the determinants of anaerobic iron oxidation, i.e., several MtoA-like multiheme cytochromes and MtrABC-like porin-cytochrome complexes which could act in the direction of electron uptake (72). The locus Ga0056053_02426–02431 is the most probable determinant of Fe(II)-oxidizing activity in *G. ferrihydriticus*, although it lacks *mtoB* and *mtoD* genes. Alternatively, Fe(II) oxidation could be determined by the homologs of the proteins of MtrABC Fe(III) reduction pathway which has recently been reported to provide inward EET in *Escherichia coli* (73). These facts highlight the requirement of further transcriptomic and proteomic evaluation of Fe(II)-oxidizing machinery of *G. ferrihydriticus*. The presence of several different electron transfer channels in the organism allows it to oxidize iron compounds in a wide redox potential window, including the potential difference of Fe^0^/Fe^2+^ couple. If corrosion via EET-mediated Fe oxidation indeed occurs in our experiments, the question of an effective electron acceptor for microbial iron oxidation arises. In our previous work, we suggested the ability of *G. ferrihydriticus* to reduce carbonate, producing acetate as the metabolic product (40, 41). However, the absence of acetate in all the cultures with steel wires, even in trace amounts, during all the incubation period argues against the utilization of carbonate as the electron acceptor in our experiments. This fact correlates with the absence of key genes of Wood-Ljungdahl pathway in *G. ferrihydriticus* genome. In this view, protons of water represent the only possible electron acceptor for Fe^0^ oxidation in our experimental systems. Thermodynamic calculations show that Fe^0^ oxidation to green rust with protons as the electron acceptors is energetically favorable and can support the growth of *G. ferrihydriticus* under our experimental conditions. The actual ∆*G*_exp_ value for this process is calculated to be −102.6 *kJ · mole^−1^*. Alternatively, iron oxidation to siderite with protons of water could serve for energy generation by *G. ferrihydriticus* upon steel wire corrosion. This process under our experimental conditions has a Δ*G*_exp_ of −20.5 *kJ · mole^−1^*, which is much lower than the thermodynamic efficiency of green rust formation under the same conditions, but still exceeds the theoretical or experimentally determined thermodynamic limits of life, i.e., −20 *kJ · mole^−1^* (74) or up to −8 *kJ · mole^−1^* (75), respectively. Based on our experimental data and these thermodynamic calculations, we propose the mechanism of interaction between *G. ferrihydriticus* and the steel wire presented in Fig. 8. It is clear that under the experimental conditions, *G. ferrihydriticus* cells act similarly to the platinum bar in the corresponding sterile control, i.e., as a catalyst of the corrosion process accompanied by the formation of green rust and hydrogen production. To realize this scenario of interaction with Fe^0^, *G. ferrihydriticus* must employ a proton-reducing, energy-conserving membrane-associated hydrogenase, coupling exergonic electron transfer to the establishment of the chemiosmotic gradient. To fuel H_2_ evolution, redox energy should be provided by a low-potential electron donor such as reduced ferredoxin (76), which is not produced during the inward electron transfer from extracellular iron via Mto-like porin-cytochrome complexes (77), identified in *G. ferrihydriticus* genome. However, the organism possesses a homolog of the ferredoxin-reducing electron-bifurcating hydrogenase of *T. kivui* (78), which is encoded in a single cluster together with HoxYHM subunits of a cytoplasmic [NiFe] hydrogenase complex. Ferredoxin, produced by the bifurcating enzyme, could be used by membrane-bound hydrogenases encoded by closely located clusters Ga0056053_02666–69 and Ga0056053_02677–88. The catalytic NiFe subunit of the latter complex shares similarity with a proton-reducing hydrogenase capable of accepting electrons from an insoluble donor (carbon felt electrode) and using a cytochrome *c_3_* as an electron-transferring partner (62, 63). Such an enzyme could link iron oxidation to hydrogen production via cytochrome proteins in *G. ferrihydriticus*, although this proposal requires separate and thorough biochemical evaluation.

### Conclusion

Our experiments have shown that the process of MIC can be just as important under alkaline anaerobic conditions as it is under neutral conditions, and it could be enhanced by autotrophic microorganisms. *G. ferrihydriticus* actively interacts with steel wire under autotrophic conditions, oxidizing metallic iron directly by the EET mechanism with protons of water serving as the most probable inorganic electron acceptors (Fig. 8).

This is in contrast to the previously described iron corrosion mechanism via direct metal-to-microbe electron transfer in a *Geobacter* species which requires an organic electron acceptor, fumarate, to carry out the process (21). Comprehensive genome analysis of *G. ferrihydriticus* revealed the determinants of anaerobic iron corrosion coupled to hydrogen production, i.e., the genes of Mto-like electron-uptaking porin-cytochrome complexes and putative H_2_-evolving membrane-bound hydrogenases which could be involved in the establishment of a transmembrane electrochemical ion gradient. The main product of corrosion induced by *G. ferrihydriticus* is green rust, and despite the inhibitory effect of its passivating film, corrosion of steel wire in *G. ferrihydriticus* cultures is a stable process with an intensity that is several times greater than that in sterile controls. Comparison of the data on bioinduced and electrochemical corrosion, provoked by the formation of galvanic couples in the presence of a platinum bar, suggests that anaerobic alkaliphilic microorganisms capable of EET could effectively catalyze steel corrosion even in the absence of organic substrates or organic electron acceptors. Such a process should be considered when planning underground nuclear waste disposals.

## Data Availability

This paper does not report any new data sets or tools.

## SUPPLEMENTAL MATERIAL

Supplemental material aem.01848-24-s0001.docx Tables S1 to S6; Figures S1 to S4.

ASM does not own the copyrights to Supplemental Material that may be linked to, or accessed through, an article. The authors have granted ASM a non-exclusive, world-wide license to publish the Supplemental Material files. Please contact the corresponding author directly for reuse.
