# Supplementary material for: Anaerobic corrosion of steel wire by Geoalkalibacter ferrihydriticus under alkaline autotrophic conditions
Source: Appl Environ Microbiol. 2025 Mar 10;91(4):e01848-24. doi: 10.1128/aem.01848-24 (PMC12016550; doi:10.1128/aem.01848-24)
Supplement: Supplemental material — Tables S1 to S6; Figures S1 to S4. [file aem.01848-24-s0001.docx]

**SUPPLEMENTARY MATERIAL**

**ANAEROBIC CORROSION OF STEEL WIRE BY *GEOALKALIBACTER FERRIHYDRITICUS* UNDER ALKALINE AUTOTROPHIC CONDITIONS**

*Daria G. Zavarzina^a^, Natalia I. Chistyakova^b^, Jaroslav Kohout^c^, Alexandr Yu. Merkel^a^, Anna A. Perevalova^d^, Denisa Kubaniova****^c^*,** *Michail S. Chernov^e^, Evgeny N. Frolov^a^, Alexey L. Klyuev^f^, Sergey N. Gavrilov^a^*

***Supplementary Table S1.*** Gibbs free energy of formation of the substances involved in the proposed corrosion processes.

| Substance |  | ${\Delta G}_{f,298}^{0}, kJ\cdot{mole}^{-1}$[1] | ${\Delta G}_{f,298}^{0}, kJ\cdot{mole}^{-1}$[2] |
| --- | --- | --- | --- |
| $\mathrm{Fe}$ | solid | 0 | 0 |
| $H_{2}$ | gas | 0 | 0 |
| $H_{2}O$ | liquid | -237.1 | -237.14 |
| $\mathrm{HCO}_{3}^{-}$ | liquid | -586.9 | -586.85 |
| $\mathrm{OH}^{-}$ | liquid | -157.3 | -157.28 |
| $\mathrm{Fe}\mathrm{CO}_{3}$ | solid | -666.7 | -666.7 |
| $\left[ {Fe}_{4}^{\left( II \right)}{Fe}_{2}^{\left( III \right)}\left( OH \right)_{12} \right]\left[ {CO}_{3}\cdot2H_{2}O \right]$ | solid | -4064 [3, 4] * | - |

* We took this value from [3] because the authors determined it more precisely and accurately compared to [4].

References:

1. Robie R.A., Hemingway B.S. Thermodynamic properties of minerals and related substances at 298.15 K and 1 bar (10[5] pascals) pressure and at higher temperatures. R.A. Robie, B.S. Hemingway, 1995.

2. Lange N.A., Dean J.A. Lange’s handbook of chemistry. N.A. Lange, J.A. Dean, 15th ed., New York St. Louis San Francisco [etc.]: McGraw-Hill, 1998.

3. Bourdoiseau J.A., Sabot R., Jeannin M., Termemil F., Refait Ph. Determination of standard Gibbs free energy of formation of green rusts and its application to the Fe(II–III) hydroxy-oxalate. // Colloids and Surfaces A: Physicochemical and Engineering Aspects. – 2012. – Vol. 410. – P. 72–80. DOI: 10.1016/j.colsurfa.2012.06.020.

4. Drissi S.H., Refait Ph., Abdelmoula M., Génin J.M.R. The preparation and thermodynamic properties of Fe(II)-Fe(III) hydroxide-carbonate (green rust 1); Pourbaix diagram of iron in carbonate-containing aqueous media. // Corrosion Science. – 1995. – Vol. 37. – No. 12. – P. 2025–2041. DOI: 10.1016/0010-938X(95)00096-3.

***Supplementary Table S2.*** Parameters of the Mӧssbauer subspectra of the initial steel wire sample. *I* – relative intensity, δ – isomer shift, ε – quadrupole shift, *H*_n_ – hyperfine magnetic field.

| **Subspectrum** | ***I*, %** | **δ, mm/s** | **ε, mm/s** | ***H*_n_, kOe** |
| --- | --- | --- | --- | --- |
| S1 | 12.9±0.8 | -0.012±0.010 | 0.001±0.005 | 306.0±0.5 |
| S2 | 87.1±0.8 | 0.002±0.001 | 0.002±0.001 | 330.0±0.1 |

***Supplementary Table S3***. Molecular hydrogen production in the cultures and sterile controls, mmol H_2_ per liter of liquid medium/culture.

| days | subsequent transfers of the culture with steel wires without magnetite or sulfide | | | IV^th^ transfer, synthesized magnetite added | IV^th^ transfer, sodium sulfide added | anaerobic sterile control | aerobic sterile control | anaerobic control with a platinum bar | anaerobic control with synthesized magnetite | anaerobic control with sodium sulfide |
| --- | --- | --- | --- | --- | --- | --- | --- | --- | --- | --- |
|  | II^nd^ transfer | III^rd^ transfer | IV^th^ transfer |  |  |  |  |  |  |  |
| 0 | 0.02±0.01 | 0.1±0.01 | 0.03±0.02 | 0.05±0.02 | 0.4±0.22 | 0.01 | 0.2 | 0.05±0.01 | 0.02 | 0.3±0.35 |
| 11 | 0.2±0.09 | 0.4±0.07 | 0.5±0.1 | 1.0±0.14 | 0.45±0.32 | 0.02 | 0.03 | 0.25±0.12 | 0.03 | 0.45±0.11 |
| 20 | 0.5±0.15 | 0.6±0.12 | 0.6±0.04 | 1.6±0.31 | 0.5±0.12 | 0.03 | 0.05 | 0.26±0.07 | 0.05 | 0.4±0.32 |
| 40 | 0.6±0.13 | 0.8±0.13 | 0.8±0.09 | 2.2±0.11 | 0.5±0.72 | 0 | 0.01 | 0.74±0.22 | 0.03 | 0.6±0.32 |
| 60 | 0.8±0.17 | 1.1±0.19 | 1.2±0.13 | 2.6±0.92 | 0.6±0.12 | 0 | 0.02 | 0.87±0.17 | 0.02 | 0.7±0.05 |
| 80 | 1.2±0.21 | 1.3±0.28 | 1.6±0.21 | 3.4±1.02 | 0.7±0.32 | 0.03 | 0.03 | 1.0±0.32 | 0.03 | 0.7±0.15 |
| 100 | 1.2±0.25 | 1.3±0.29 | 1.7±0.21 | 3.6±0.52 | 0.8±1.02 | 0.02 | 0 | 1.65±0.07 | 0.01 | 0.9±1.22 |
| 130 | 1.4±0.39 | 1.6±0.45 | 2.2±0.38 | 4.0±0.32 | 0.7±0.12 | 0 | 0 | 1.95±0.24 | 0.03 | 0.9±0.53 |
| 160 | 5.4±0.53 | 5.4±0.15 | 5.9±0.69 | 5.4±0.12 | 0.9±0.34 | 0.01 | 0.03 | 2.48±0.18 | 0.02 | 1.2±0.21 |
| 180 | 5.5±0.52 | 6.0±0.15 | 6.5±0.24 | 5.8±0.42 | 1.4±0.56 | 0.03 | 0.02 | 3.43±0.07 | 0.02 | 1.6±0.36 |

All experiments were performed in triplicates, mean values with standard deviations are presented where appropriate.

| **Control** | **Cations** | ***I* (%)** | **δ, mm/s** | **ε, mm/s** | ***H*_n_, kOe** |
| --- | --- | --- | --- | --- | --- |
| anaerobic sterile control | Fe^0^ (metal iron) | 85.3±0.7 | 0.0243±0.0004 | 0.0017±0.0004 | 329.7±0.1 |
|  | Fe^0^ (metal iron) | 13.5±0.6 | 0.024±0.004 | 0.0022±0.0035 | 306.3±0.4 |
|  | Fe^2+^(green rust) | 0.38±0.17 | 1.022±0.17 | 0.87±0.18 |  |
|  | Fe^3+^(green rust) | 0.88±0.31 | 0.26±0.12 | 0.13±0.19 |  |
| aerobic sterile control | Fe^0^ (metal iron) | 76.9±0.7 | 0.0041±0.0005 | 0.0003±0.0005 | 329.8±0.1 |
|  | Fe^0^ (metal iron) | 14.4±0.8 | 0.013±0.004 | 0.010±0.004 | 307.0±0.1 |
|  | Fe^2+^ (green rust) | 5.4±0.3 | 1.20±0.01 | 1.00±0.01 |  |
|  | Fe^3+^ (green rust) | 3.3±0.3 | 0.17±0.02 | 0.34±0.02 |  |
| anaerobic control with a platinum bar | Fe^0^ (metal iron) | 71.8±0.7 | 0.0019±0.0011 | 0.0002±0.0011 | 329.9±0.1 |
|  | Fe^0^ (metal iron) | 11.4±0.6 | -0.024±0.009 | 0.003±0.009 | 306.8±0.8 |
|  | Fe^2+^ (green rust) | 6.7±1.1 | 1.23±0.04 | 1.12±0.04 |  |
|  | Fe^3+^ (green rust) | 10.2±1.1 | 0.33±0.03 | 0.32±0.03 |  |
| anaerobic control with sodium sulfide | Fe^0^ (metal iron) | 78.8±0.7 | 0.0239±0.0005 | 0.0021±0.0005 | 329.5±0.1 |
|  | Fe^0^ (metal iron) | 11.1±0.6 | 0.022±0.004 | 0.004±0.004 | 305.9±0.4 |
|  | Fe^2+^ (green rust) | 3.8±0.4 | 1.22±0.04 | 1.26±0.04 |  |
|  | Fe^3+^ (green rust) | 6.4±0.4 | 0.32±0.04 | 0.37±0.04 |  |
| IV^th^ transfer of the culture supplemented with sodium sulfide | Fe^0^ (metal iron) | 76.5±0.7 | 0.0233±0.0005 | 0.0010±0.0005 | 329.7±0.1 |
|  | Fe^0^ (metal iron) | 12.6±0.7 | 0.022±0.004 | 0.002±0.004 | 307.3±0.5 |
|  | Fe^2+^ (green rust) | 3.7±0.3 | 1.17±0.03 | 1.25±0.04 |  |
|  | Fe^3+^ (green rust) | 7.2±0.4 | 0.329±0.030 | 0.400±0.028 |  |
| IV^th^ transfer of the culture supplemented with synthesized magnetite | Fe^0^ (metal iron) | 78.1±0.9 | 0.0237±0.0005 | 0.0011±0.0005 | 329.8±0.1 |
|  | Fe^0^ (metal iron) | 12.1±0.9 | 0.012±0.005 | -0.003±0.005 | 307.0±0.6 |
|  | Fe^2+^ (green rust) | 4.7±0.3 | 1.23±0.01 | 1.10±0.01 |  |
|  | Fe^3+^ (green rust) | 5.14±0.26 | 0.21±0.02 | 0.33±0.01 |  |

***Supplementary Table S4.*** Parameters of Mӧssbauer subspectra of iron cations and atoms in newly formed mineral phases (precipitates) and in the steel wire after 180 days of incubation of sterile controls and control cultures supplemented with magnetite or sodium sulfide.

Mean of triplicates are presented. *I* – relative intensity, *δ* – isomer shift, *ε* – quadrupole shift.

***Supplementary Table S5.*** Parameters of Mӧssbauer subspectra corresponding to iron atoms and cations in newly formed phases, the steel wire and precipitates in the 1^st^ and 4^th^ transfers of *G. ferrihydriticus* cultures. Means of triplicates are shown. *I* – relative intensity, δ – isomer shift, ε – quadrupole shift; *H*_n_ – hyperfine magnetic field.

| **Sample/culture** | **Cations** | ***I*, %** | **δ, mm/s** | **ε, mm/s** | ***H*_n_, kOe** |
| --- | --- | --- | --- | --- | --- |
| Transformed wire  I^st^ transfer culture, 120 days of incubation | Fe^0^ (metal iron) | 82.0±0.4 | 0.0014±0.0005 | 0.0016±0.0005 | 330.5±0.1 |
|  | Fe^0^ (metal iron) | 9.2±0.3 | 0.010±0.005 | 0.005±0.005 | 306.0±0.5 |
|  | Fe^2+^ (green rust) | 2.8±0.5 | 1.21±0.04 | 1.14±0.03 |  |
|  | Fe^3+^ (green rust) | 6.1±0.5 | 0.30±0.02 | 0.39±0.02 |  |
| Precipitate  I^st^ transfer culture, 120 days of incubation | Fe^0^ (metal iron) | 11.0±0.4 | 0.00 | 0.00 | 330.1 |
|  | Fe^0^ (metal iron) | 1.2±0.5 | 0.00 | 0.00 | 306.5 |
|  | Fe^2+^ (green rust) | 47.8±1.0 | 1.15±0.01 | 1.38±0.01 |  |
|  | Fe^3+^ (green rust) | 20.0±0.6 | 0.35±0.01 | 0.34±0.01 |  |
|  | Fe^2+^ (siderite) | 20.0±1.0 | 1.22±0.01 | 0.92±0.01 |  |
| Transformed wire  IV^th^ transfer culture, 180 days of incubation | Fe^0^ (metal iron) | 81.7±0.4 | 0.00 | 0.00 | 330.0 |
|  | Fe^0^ (metal iron) | 12.2±0.1 | 0.00 | 0.00 | 306.1 |
|  | Fe^2+^ (green rust) | 2.3±0.4 | 1.215 | 1.17±0.04 |  |
|  | Fe^3+^ (green rust) | 3.9±0.4 | 0.26±0.03 | 0.47±0.02 |  |
| Precipitate  IV^th^ transfer culture, 180 days of incubation | Fe^0^ (metal iron) | 28.1±0.9 | 0.003±0.004 | 0.003±0.004 | 329.9±0.4 |
|  | Fe^0^ (metal iron) | 2.8±0.8 | 0.00 | 0.00 | 311±4 |
|  | Fe^2+^ (green rust) | 16.7±1.2 | 1.280 | 1.18±0.02 |  |
|  | Fe^3+^ (green rust) | 52.4±1.1 | 0.34±0.01 | 0.44±0.01 |  |

***Supplementary Table S6*.** Cell counting based on the results of qPCR quantification of 16S rRNA gene copies of *G. ferrihydriticus* Z-0531 (means of triplicates with standard deviations are shown).

| **Transfer of the culture with the steel sponge** | **Cells per ml^-1^ of the culture by the end of incubation** |
| --- | --- |
| II^nd^ | 9.0±0.8×10^5^ |
| III^rd^ | 2.6±1.3×10^6^ |
| IV^th^ | 1.9±1.0×10^6^ |

***Supplementary Figure S1.*** XRF spectra of the tested steel wire sponge.

***Supplementary Figure S2.*** XRD spectra of the steel wire (a) before and (b) after bacterial growth and (c) the precipitate formed after bacterial growth of the 1^st^ transfer culture
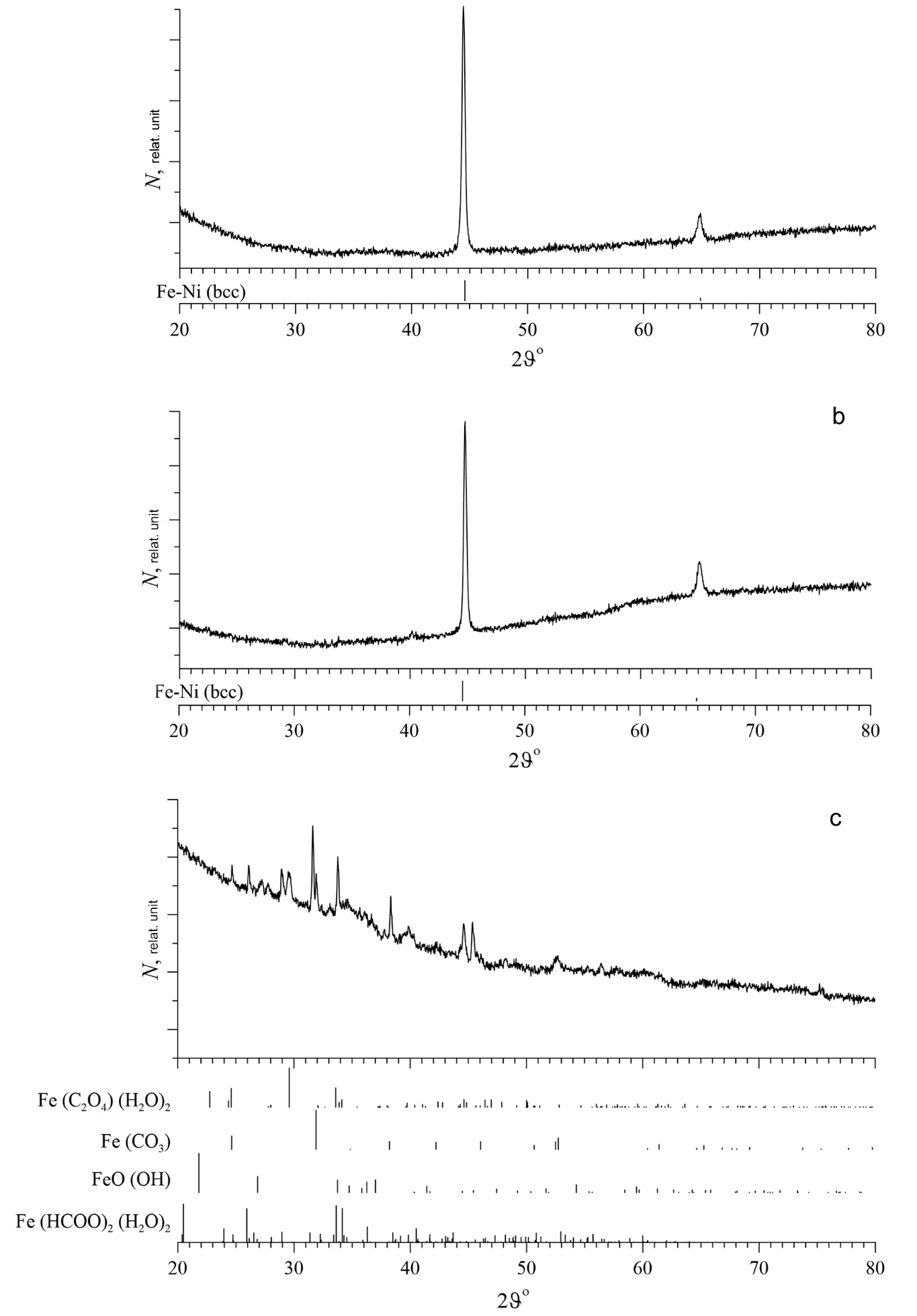
.

a

***Supplementary Figure S3.*** Steel wire and mineral precipitate after 180 days of incubation at 37^0^C: (a) aerobic sterile control; (b) anerobic sterile control with a platinum bar; (c) culture supplemented with synthesized magnetite; (d) control supplemented with synthesized magnetite; (e) culture supplemented with sodium sulfide; (f) control supplemented with sodium sulfide. Arrows on (c), (e), and (f) point to H_2_ bubbles.


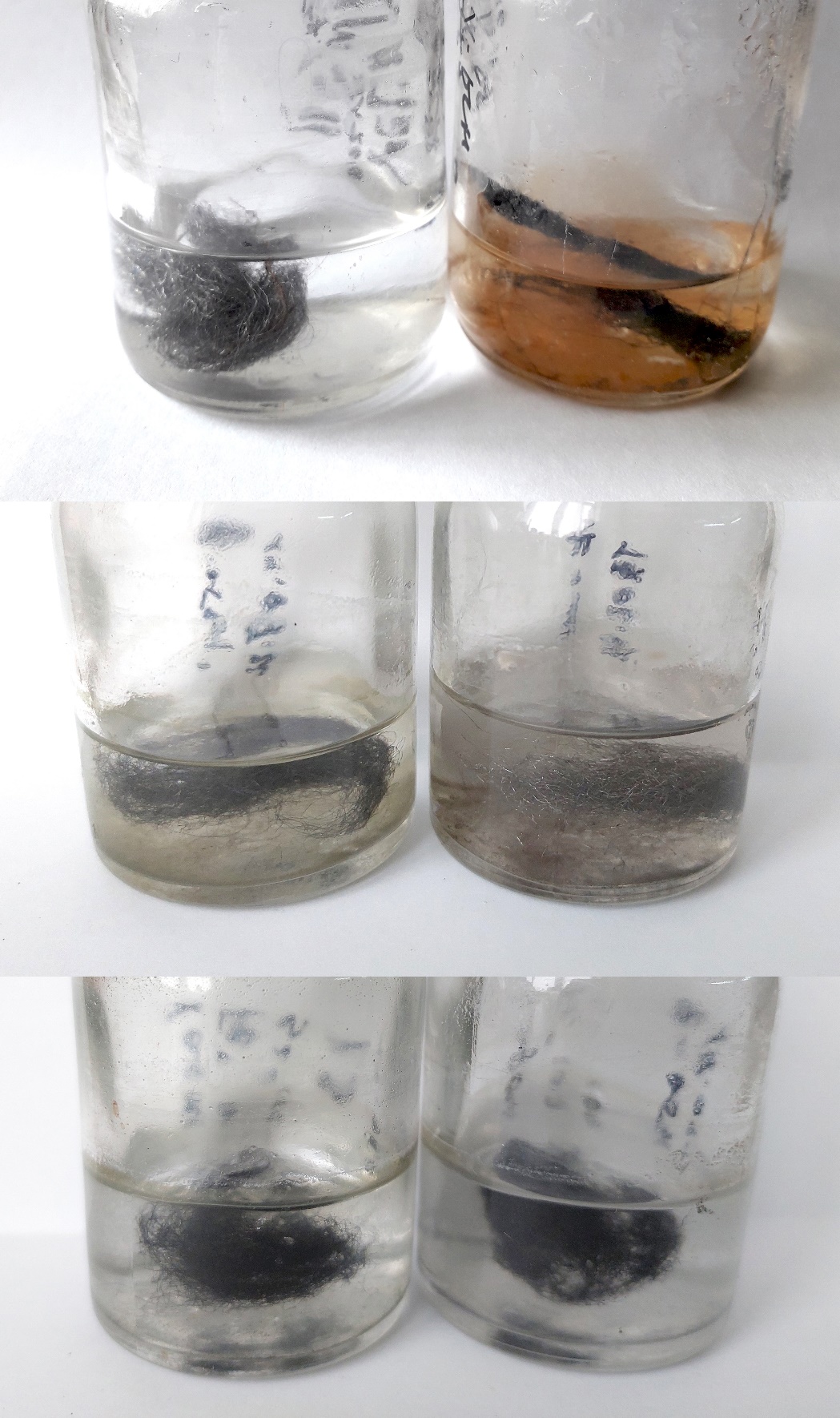


a

b

c

d

e

f


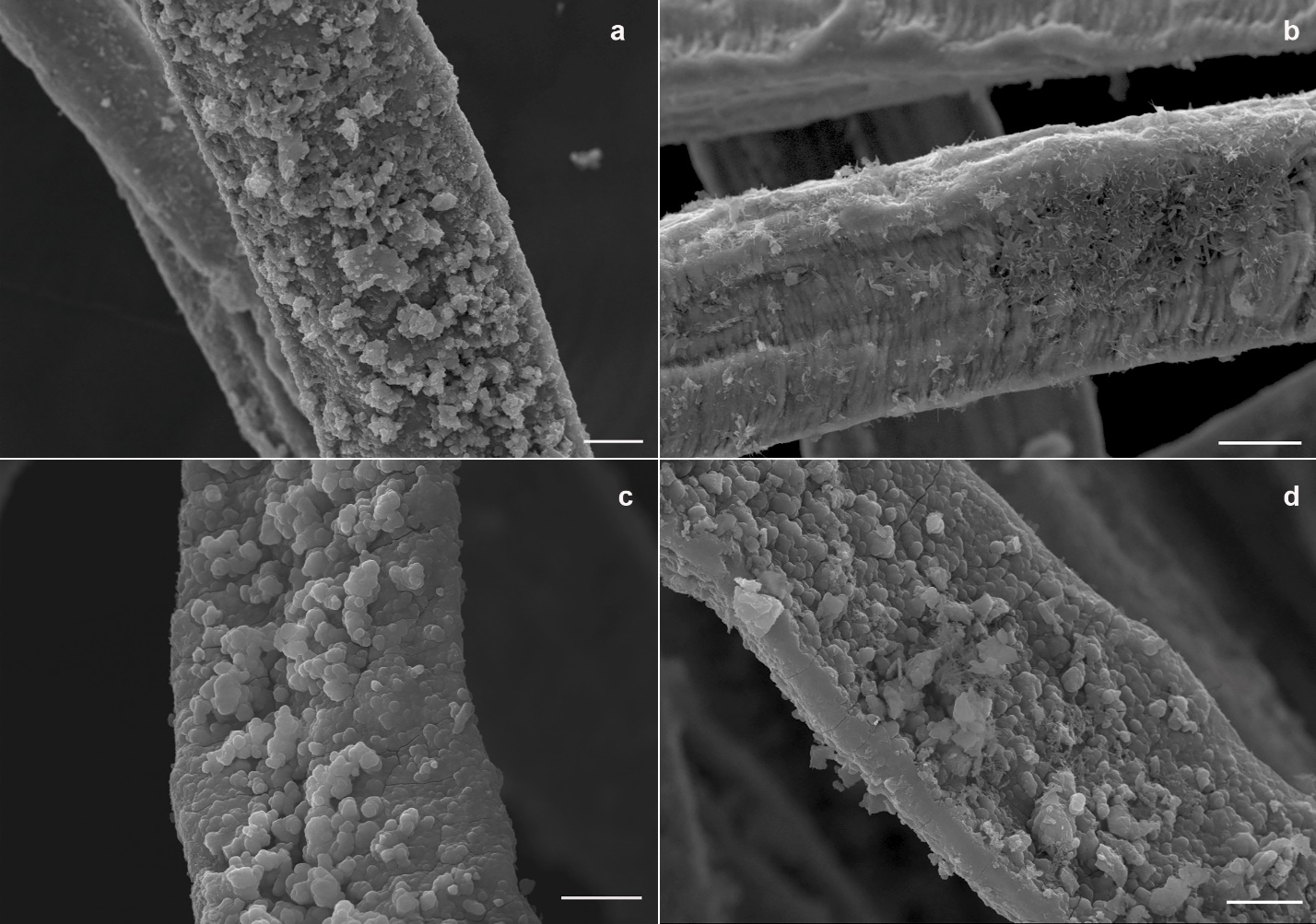
***Supplementary Figure S4.*** SEM micrographs of the steel wire after 180 days of incubation in *G. ferrihydriticus* culture with the addition of magnetite (a-b) and sodium sulfide (c-d): (a, c) control experiments; (b, d) sterile controls.
